# Observation of Near‐Infrared Photothermal and Photoacoustic Effects in a Metallosupramolecular Trefoil Knot

**DOI:** 10.1002/anie.202518415

**Published:** 2025-10-03

**Authors:** Ye Lu, Zhi‐Jie Li, Yi Li, Lu An, Shi‐Ping Yang, F. Ekkehardt Hahn

**Affiliations:** ^1^ The Education Ministry Key Lab of Resource Chemistry, Joint International Research Laboratory of Resource Chemistry of Ministry of Education, Shanghai Frontiers Science Center of Biomimetic Catalysis, College of Chemistry and Materials Science Shanghai Normal University Shanghai 200234 P.R. China; ^2^ Institut für Anorganische und Analytische Chemie Universität Münster Corrensstrasse 30 48149 Münster Germany

**Keywords:** Bisimidazole, Half‐sandwich complexes, Phothermal effect, Photoacoustic effect, Trefoil knots

## Abstract

Coordination‐driven self‐assembly is a facile and powerful strategy for the construction of metallosupramolecular mechanically interlocked molecules (MIMs). However, the limited stability of most MIMs constitutes a challenge with respect to the evaluation of their practical applications. Herein, we demonstrate a rational strategy to control the self‐assembly outcome for selected metallosupramolecular structures. The reaction of a biphenyl‐bridged bisimidazole ligand with dinuclear building blocks of varying lengths leads, depending on the size of the dinuclear building block, to a simple [1+1] metallacycle, a twisted [2+2] three‐dimensional trapezoidal structure, or an intricate [3+3] metallosupramolecular trefoil knot. The trefoil knot exhibits exceptional structural stability, resisting decomposition upon dilution in polar solvent. Its knotted topology enforces strong intramolecular π⋅⋅⋅π stacking interactions, which red‐shift the absorption band into the near‐infrared (NIR) region. Benefiting from its stability and NIR absorption, the trefoil knot displays a high photothermal conversion efficiency (*η* = 73.5%) and a potent photoacoustic (PA) effect in water, even at low concentrations.

## Graphical abstract

A metallosupramolecular trifold knot has been prepared from three bisimidazole ligands and three dinuclear half‐sandwich metal complexes. The topology of the trifold knot enforces strong π⋅⋅⋅π interactions between the building blocks, which results in near‐infrared (NIR) light absorption. This leads to an efficient photothermal conversion and a remarkable photoacoustic (PA) response, while the trifold knot exhibits a high stability.

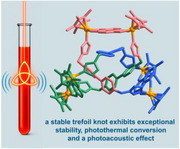

## Introduction

Coordination‐driven self‐assembly has been widely employed and proven to be an efficient and convenient strategy for the construction of mechanically interlocked molecules (MIMs).^[^ ^1^, ^2^, ^3^, ^4^ ^]^ A large number of metallosupramolecular catenanes utilizing Werner‐type ligands, as well as organometallic catenanes based on N‐heterocyclic carbene ligands, have been synthesized utilizing this method including, [2], [3], and [4]catenanes,^[^ ^5^, ^6^, ^7^, ^8^, ^9^, ^10^, ^11^, ^12^ ^]^ Borromean rings,^[^ ^13^, ^14^, ^15^ ^]^ Solomon links,^[^ ^16^, ^17^, ^18^ ^]^ and related derivatives.^[^ ^19^, ^20^, ^21^, ^22^, ^23^, ^24^ ^]^ Coordination‐driven self‐assembly, while straightforward and high‐yielding, enables the one‐step construction of complex topological structures. However, the resulting MIM structures often exhibit limited structural stability in solution, as, for example, a dynamic equilibrium between the [*n*]catenanes and their monoring components exists.^[^ ^25^ ^]^ Although this equilibrium has attracted considerable interest from chemists and has been utilized in applications such as separation, it undeniably poses challenges for the study and further application of metallosupramolecular MIMs in solution.^[^ ^26^, ^27^ ^]^ The same stability issue applies to trefoil knots, a type of mechanically interlocked molecular architecture consisting of three crossing points and a closed‐loop configuration resembling a cloverleaf.^[^ ^28^, ^29^ ^]^ As one of the simplest nontrivial knots, trefoil knots have garnered significant attention in supramolecular chemistry due to their intriguing topology and potential applications in molecular machines, catalysis, and spin filters.^[^ ^30^, ^31^, ^32^, ^33^, ^34^, ^35^ ^]^ However, for metallosupramolecular trefoil knots, the detailed nuclear magnetic resonance (NMR) characterization and potential applications remain significantly limited, primarily due to their insufficient structural stability in solution.^[^ ^36^, ^37^ ^]^

Utilizing the quenching effect on luminescence observed for the half‐sandwich metal structures^[^ ^38^, ^39^ ^]^ and the red‐shifted absorption induced by donor–acceptor interactions, heterogeneous half‐sandwich Cp*Rh (Cp* = pentamethylcyclopentadienyl) [2]catenanes with photothermal conversion capabilities have been prepared.^[^ ^40^ ^]^ However, due to a dynamic equilibrium in solution, the [2]catenanes decomposed into simple rings upon dilution. As a result, their photothermal conversion performance could only be investigated in the solid state, limiting the evaluation and application of this property in solution.^[^ ^41^, ^42^ ^]^

While π⋅⋅⋅π stacking interactions can indeed result in red‐shifted absorption bands, particularly in donor–acceptor systems, this effect is often not sufficient to achieve near‐infrared (NIR) absorption.^[^ ^43^, ^44^, ^45^ ^]^ Interestingly, certain metal‐to‐ligand charge transfer (MLCT) transitions between selected ligands and half‐sandwich Cp*‐Rh or cymene‐Ru units can enable absorption in the red‐light region, which is very close to the near‐infrared region (NIR), resulting in green solutions of the respective derivatives.^[^ ^46^ ^]^ We therefore assumed that the combination of MLCT transitions and π⋅⋅⋅π stacking in one complex may provide a pathway to achieve NIR absorption.

The photoacoustic (PA) effect is a phenomenon wherein absorbed light energy is converted into heat, leading to enhanced thermal vibrations of molecules in their local microenvironment and the subsequent generation of ultrasonic waves.^[^ ^47^ ^]^ This effect has been widely exploited, most notably for contrast‐enhanced PA imaging.^[^ ^48^ ^]^ Given that the PA effect is fundamentally a derivative of photothermal conversion, it is hypothesized that half‐sandwich metal‐derived catenanes exhibiting photothermal properties should also possess a significant PA response. However, to our knowledge, this subject has been rarely explored. Unlike photothermal conversion, which typically employs continuous wave lasers, the PA effect relies on pulsed lasers to induce rapid and localized heating, generating a sufficiently strong transient temperature gradient to produce detectable ultrasound signals.^[^ ^49^ ^]^ This distinction highlights the need to study suitable compounds in solution, particularly under a low concentration regimen, where the structural stability of the molecules in solution plays a critical role.^[^ ^50^ ^]^

Herein, we report a stable metallosupramolecular trefoil knot exhibiting exceptional NIR PA effects in aqueous solution. Different linear dinuclear half‐sandwich metal complexes and a bisimidazole ligand were used to generate polynuclear assemblies. While the longest dinuclear complex yielded with the bisimidazole a [1+1] organometallic trapezoid, the shortest one resulted in the formation of a twisted three‐dimensional metallosupramolecular trapezoidal structure. By selecting a midsize dinuclear half‐sandwich Cp*‐Rh (Cp* = pentamethylcyclopenadienyl) complex as a building block, the reaction with the bisimidazole yielded an intricate [3+3] metallosupramolecular trefoil knot. This trefoil knot exhibited excellent stability in solution, being resistant to dilution and low‐polarity solvents. Moreover, the formation of the trefoil knot induced π⋅⋅⋅π stacking interactions between the dinuclear metal complex and the bisimidazole ligands, resulting in a red‐shift of the absorption band into the NIR region. Based on its remarkable stability and NIR absorption ability, the trefoil knot featured an outstanding NIR photothermal conversion capability in aqueous solutions and, more importantly, an excellent NIR PA effect, even at low concentrations.

## Results and Discussion

**Scheme 1:**
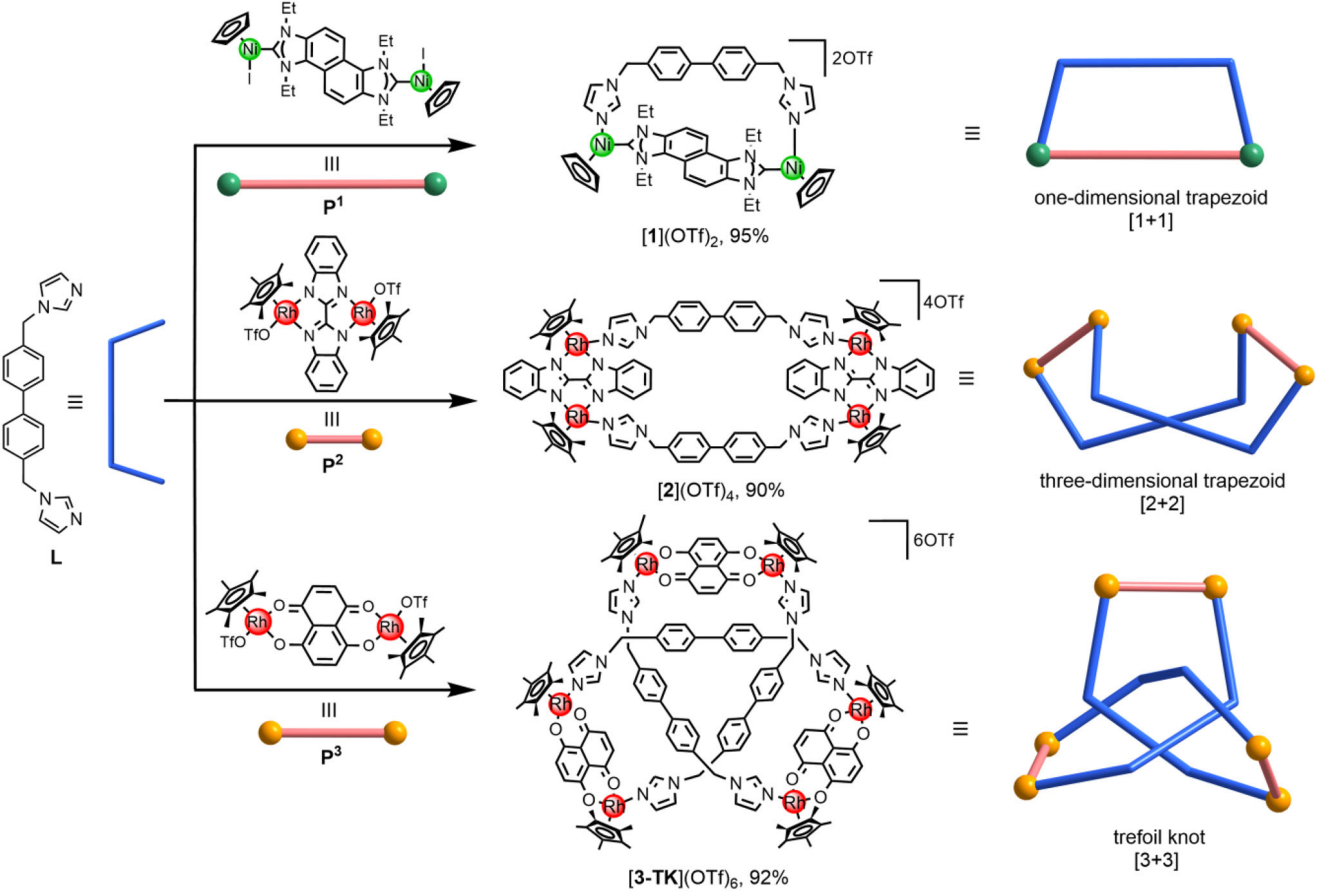
Synthesis of the [1+1] adduct [**1**](CF_3_SO_3_)_2_, the [2+2] trapezoid [**2**](CF_3_SO_3_)_4_ and the [3+3] trefoil knot [**3‐TK**](CF_3_SO_3_)_6_ obtained from the dinuclear metal complexes **P^1^**–**P^3^** and bisimidazole ligand **L**.

Bipyridyl or polypyridyl ligands have been widely employed in coordination‐driven self‐assembly for the construction of MIMs, while the use of bisimidazole ligand remains comparatively underexplored^[^ ^51^, ^52^ ^]^ The imidazole group contains two heteroatoms, which can be regarded as one pyrrole‐like nitrogen and one pyridine‐like nitrogen. The pyrrole‐like nitrogen atom is readily alkylated with alkyl halides, leaving the pyridine‐like nitrogen atom available for metal coordination. The N‐alkylated imidazole group is a five‐membered ring, featuring, after coordination to a metal center, an intrinsic angle between the N–alkyl bond and the N–metal bond.^[^ ^53^ ^]^ This angular geometry may provide a structural advantage for the construction of intricate interlocked structures. To explore this potential, we selected a biphenyl‐bridged bisimidazole ligand **L** (**L** = 4,4′‐bis((1*H*‐imidazol‐1‐yl)methyl)‐1,1′‐biphenyl), in which the imidazole rings are connected to the biphenyl group via flexible methylene linkers, leaving the unsubstituted nitrogen atoms accessible for metal coordination (Scheme 1).

In recent years, we have studied the self‐assembly of metallosupramolecular interlocked structures using preorganized dinuclear Cp‐Ni precursors, bridged by di‐NHC ligands (NHC = *N*‐heterocyclic carbene) and additional bipyridyl ligands.^[^ ^10^, ^13^ ^]^ As an extension of these studies, we herein investigate the reaction of related dinuclear complexes with the aforementioned bisimidazole ligand **L**.

The reaction of 1.0 equiv. of the benzobiscarbene complex **P^1^** with 1.0 equiv. of the bisimidazole ligand **L** and 2.0 equiv. of AgOTf in methanol (Scheme 1) yielded the [1+1] adduct [**1**](CF_3_SO_3_)_2_ in 95% yield, as confirmed by NMR spectroscopy (Figures S1–S5) and electrospray ionization mass spectrometry (ESI‐MS spectrometry, Figures S6 and S7). The ^1^H NMR spectrum of [**1**](CF_3_SO_3_)_2_ displayed signals corresponding to a 1:1 stoichiometric ratio of the dinuclear complex **P^1^** and ligand **L**. In addition, the ESI‐MS spectrum clearly indicated that dication [**1**]^2+^ is a [1+1] structure composed of one dinuclear metal complex and one bisimidazole ligand. The ESI‐MS spectrum featured prominent peaks at *m/z* = 1029.2544 (calcd. for [**1**+OTf]^+^, 1029.2537) and 440.1591 (calcd. for [**1**]^2+^,440.1505) with matching isotope distribution (Figures S6 and S7).

**Figure 1:**
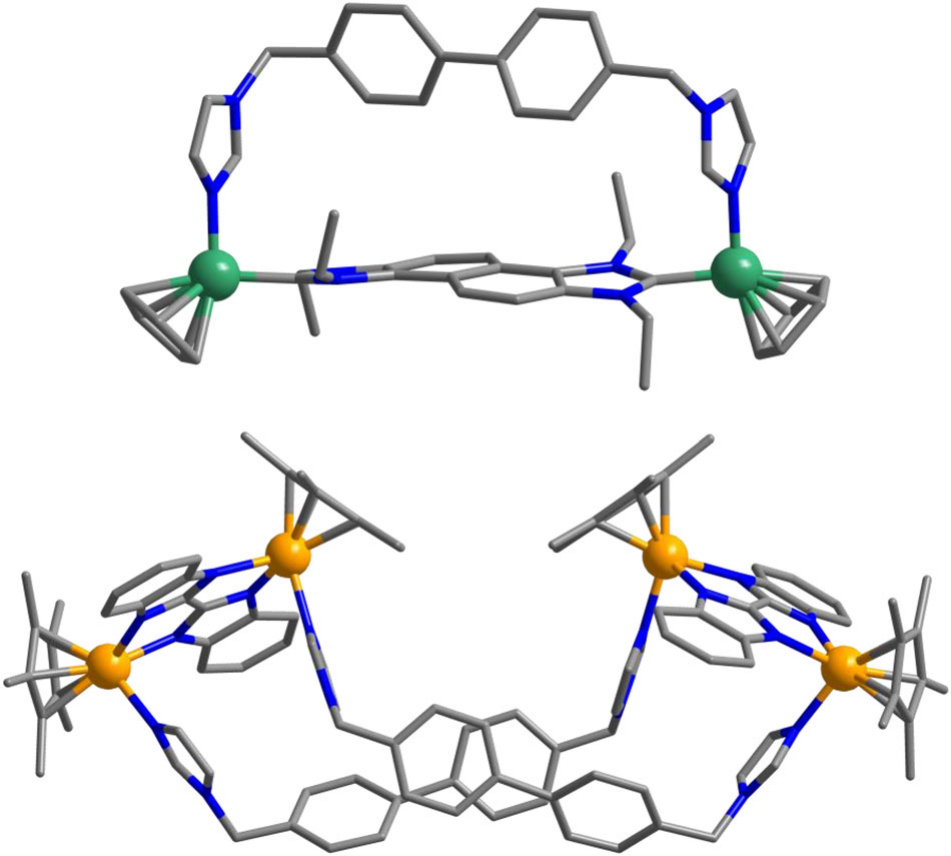
Molecular structures of the dinuclear cation [**1**]^2+^ in [**1**](CF_3_SO_3_)_2_⋅MeOH (top) and of the tetranuclear cation [2]^4+^ in [**2**](CF_3_SO_3_)_4_⋅4MeOH⋅4H_2_O (bottom, color code: C, grey; N, blue; Ni, green; Rh, yellow).

Green crystals of composition [**1**](CF_3_SO_3_)_2_⋅MeOH suitable for an X‐ray diffraction analysis were obtained by the slow diffusion of diethyl ether into a methanol/DMSO (10:1 v/v) solution of the compound. Single‐crystal X‐ray diffraction (SC‐XRD) analysis unequivocally established the molecular structure of [**1**]^2+^ as a discrete [1+1] adduct of **P^1^** and **L** (Figure 1, top).^[^ ^54^ ^]^ The distance between the two coordinating nitrogen atoms of ligand **L** (11.944 Å) closely matches the Ni···Ni distance in **P^1^** (11.937 Å), which is likely a key factor for the formation of the [1+1] adduct (Figure S36).

In order to disrupt the geometric congruence observed in dication [**1**]^2+^ and to construct alternative structures with ligand **L**, the dinuclear Cp*‐Rh complex **P^2^**, featuring a much shorter metal‐to‐metal separation of only 5.6 Å, was selected. The reaction of **P^2^** with ligand **L** in methanol under the same conditions that were employed for the synthesis of [**1**](CF_3_SO_3_)_2_ but without the addition of AgOTf resulted in the formation of [**2**](CF_3_SO_3_)_4_ (90% yield), a [2+2] macrocycle adopting a twisted, three‐dimensional trapezoidal structure (Scheme 1). The ^1^H NMR spectrum of [**2**](CF_3_SO_3_)_4_ displayed signals consistent with a 1:1 ratio of precursor **P^2^** and ligand **L** (Figures S8−S12). However, the resonances of the protons of the imidazole backbone in [**2**]^4+^ (*δ* = 6.00 and 5.33 ppm, Figure S8) are shifted significantly upfield compared to the equivalent resonances in [**1**]^2+^ (*δ* = 7.32 and 7.07 ppm, Figure S1). The ESI‐MS spectrum revealed a tetranuclear [2+2] composition of tetracation [**2**]^4+^ with the most intense peaks observed at *m/z* = 1171.2257 (calcd. for [**2**+2OTf]^2+^, 1171.2252) and 731.4446 (calcd. for [**2**+OTf]^3+^, 731.5003) with perfectly matching isotope distribution (Figures S13 and S14).

Orange crystals of [**2**](CF_3_SO_3_)_4_⋅4MeOH⋅4H_2_O were obtained in high yield by the slow diffusion of diethyl ether into a methanol/DMSO (5:1 v/v) solution of the compound. SC‐XRD analysis confirmed that complex cation [**2**]^4+^ is a tetranuclear [2+2] structure composed of two dinuclear metal complexes **P^2^** bridged by two ligands **L** (Figure 1, bottom).^[^ ^54^ ^]^ The overall structure is best described as a trapezoidal prism (Figure 1, bottom). Owing to the configuration and flexibility of the bisimidazole ligands, the macrocycle exhibits significant twisting. If the short edges, defined by the **P^2^** complexes, are conceptually rotated to align them in parallel, a three‐dimensional trapezoidal prism can be visualized (Figure S37). The distance between two rhodium atoms of the same **P^2^** unit measures only 5.6 Å, which forces two bisimidazole ligands closer together, resulting in a separation between them of only 3.7 Å. This geometric situation explains the upfield shift observed for the imidazole backbone protons in the ^1^H NMR spectrum (Figures S8 and S38). The absorption spectrum of [**2**](CF_3_SO_3_)_4_ is shown in Figure S26.

**Figure 2:**
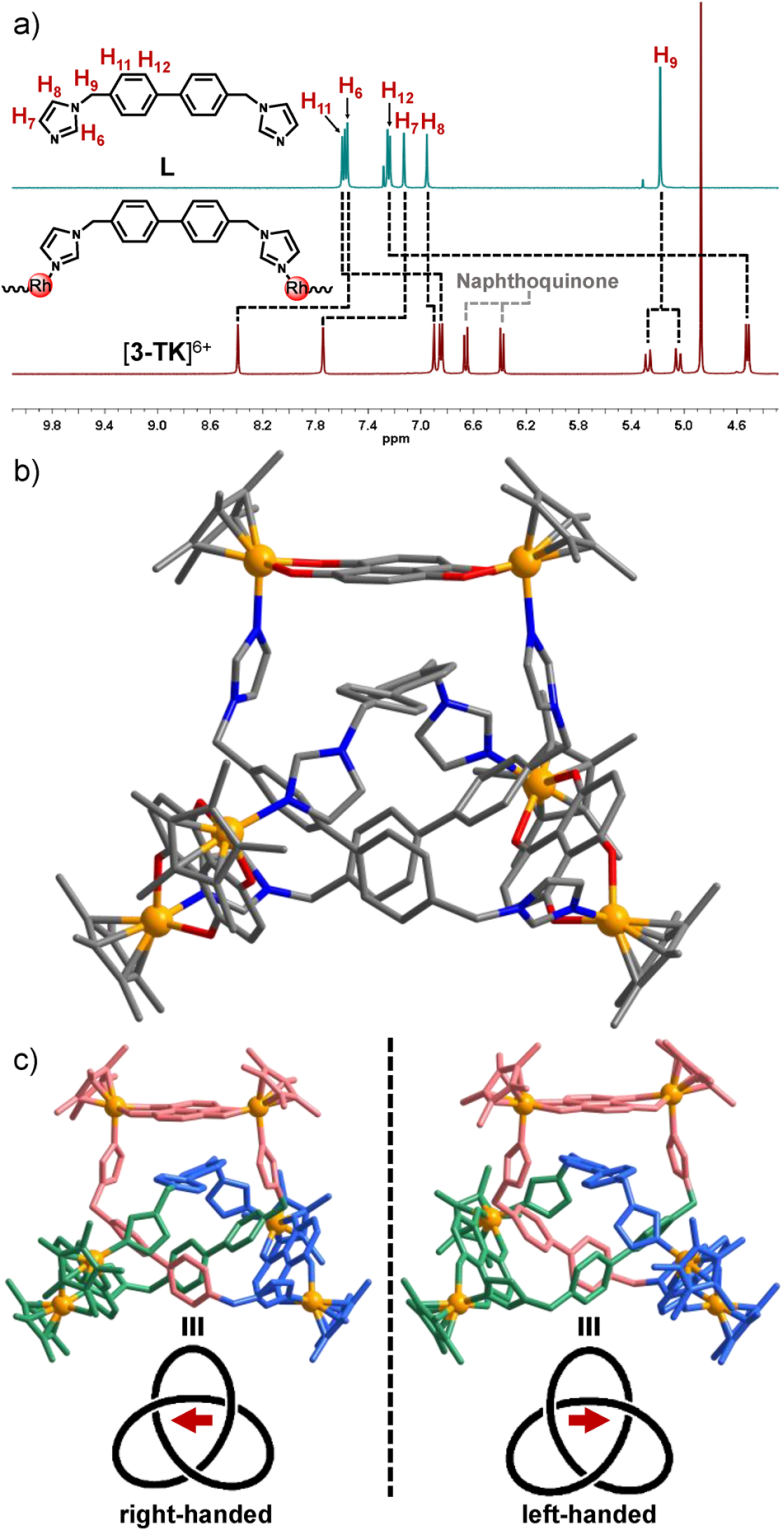
a) Partial ^1^H NMR spectra of free ligand **L** (in CDCl_3_, [20.0 mM], 400 MHz) and trefoil knot [**3‐TK**](CF_3_SO_3_)_6_ (in CD_3_OD, [13.0 mM], 400 MHz). b) Molecular structure of trefoil knot cation [**3‐TK**]^6+^ (color code: C, grey; N, blue; Ni, green; Rh, yellow; hydrogen atoms are omitted). c) The right‐handed and left‐handed enantiomers, with each leaf of the trefoil knot depicted in a different color (hydrogen atoms are omitted).

Since the previously used dinuclear metal complexes are either too long or too short to form an intricate structure with ligand **L**, we next selected a metal complex with intermediate size for the synthesis of an interlocked structure. Metal complex **P^3^** was chosen (Scheme 1), not only because its intramolecular metal‐to‐metal distance measures a moderate 8.3 Å, but also because the aromatic surface of its naphthalene bridge could facilitate crucial π⋅⋅⋅π stacking interactions. Stirring of a mixture of precursor **P^3^** (1.0 equiv.) and bisimidazol ligand **L** (1.0 equiv.) in CD_3_OD for 12 h at ambient temperature resulted in a clear green solution. The ^1^H NMR spectrum of the solution [13.0 mM] indicated the formation of a single, highly symmetric compound (Figure S15), which we subsequently isolated in 92% yield and identified as the trefoil knot [**3‐TK**](CF_3_SO_3_)_6_ (Scheme 1). This structural assignment is based on a comprehensive set of NMR experiments, including ^13^C, DEPT‐135, ^1^H‐^1^H ROESY, ^1^H‐^1^H COSY, ^1^H‐^13^C HSQC, and ^1^H‐^13^C HMBC NMR spectroscopy (Figures S16–S21). Differently from the situation in the free bisimidazole, selected protons of ligand **L** in cation [**3‐TK**]^3+^ experience strong shielding (Figures 2a and S15). For example, the resonance of the aromatic protons H_11_ of the biphenyl group shifted from *δ* = 7.57 (for free ligand **L**) to *δ* = 6.85 (for [**3‐TK**]^3+^). An even stronger upfield shift was observed for the second phenyl proton H_12_ (*δ* = 7.23 for free ligand **L**, *δ* = 4.52 for [**3‐TK**]^3+^). In addition, the resonance for the methylene protons H_9_ split into two doublets (Figure 2a) due to the diastereotopic nature of these protons in cation [**3‐TK**]^3+^. The observation of the strong shielding effects and of the diastereotopic protons H_9_ are characteristic features of a mechanically interlocked structure stabilized by strong π⋅⋅⋅π stacking interactions. Furthermore, ESI mass spectrometry analysis provided confirmation of the [3+3] constitution of [**3‐TK**]^3+^, assembled from three metal complexes **P^3^** and three ligands **L**. The spectrum was dominated by intense peaks at *m/z* = 1766.4950 (calcd. for [**3‐TK**+4OTf]^2+^ 1766.2208) and 808.4334 (calcd. for [**3‐TK**+2OTf]^4+^ 808.3836), both featuring the correct isotope distribution (Figures S24 and S25).

**Figure 3:**
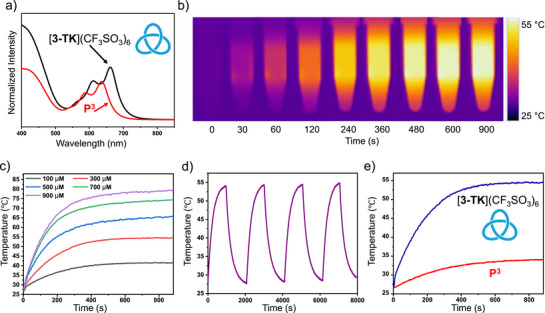
a) Partial absorption spectra of [**3‐TK**](CF_3_SO_3_)_6_ and of dinuclear metal complex **P^3^** in methanol. b) IR thermal images of aqueous solutions of [**3‐TK**](CF_3_SO_3_)_6_ (300 µM, with 1% DMSO) under 730 nm photoirradiation. c) Photothermal conversion curves of aqueous solutions of [**3‐TK**](CF_3_SO_3_)_6_ of different concentrations (with 1% DMSO) upon 730 nm laser photoirradiation (1.0 W cm^−2^). d) Photothermal stability of [**3‐TK**](CF_3_SO_3_)_6_ aqueous solutions (300 µM, with 1% DMSO) after four laser on/off switching cycles. e) Comparison of the heating curve of aqueous solutions of [**3‐TK**](CF_3_SO_3_)_6_ (300 µM, with 1% DMSO) and dinuclear complex **P^3^** (900 µM, with 1% DMSO).

Fortunately, green block crystals of [**3‐TK**](CF_3_SO_3_)_6_⋅2DMSO⋅5MeOH⋅3H_2_O suitable for an X‐ray diffraction study were isolated in high yield via slow diffusion of diethyl ether into a methanol/DMSO (10:1 v/v) solution of the compound. The single‐crystal X‐ray analysis unequivocally confirmed that cation [**3‐TK**]^6+^ adopts a discrete trefoil knot topology (Figure 2b).^[^ ^54^ ^]^ In this intricate architecture, three imidazolyl ligands **L** bridge three dinuclear complexes **P^3^**, with the ligands weaving through each other to form a hexanuclear [3+3] assembly. The molecular structure further reveals that the naphthalene planes of the dinuclear complexes are stacked with the biphenyl groups of the ligands **L**. The distance between these stacked planes measures only 3.4 Å, a value characteristic of strong π⋅⋅⋅π stacking interactions (Figures 2b and S39). These π⋅⋅⋅π interactions are fully consistent with the remarkable upfield shifts observed for the biphenyl protons in the ^1^H NMR spectrum of [**3‐TK**](CF_3_SO_3_)_6_ (Figure 2a). Furthermore, the strong π⋅⋅⋅π interactions induce a significant red‐shift in the absorption spectrum, from *λ*_max_ ≈ 630 nm for complex **P^3^** to *λ*_max_ ≈ 680 nm for [**3‐TK**](CF_3_SO_3_)_6_, with the absorption tail extending into the near‐infrared region beyond 700 nm (Figures 3a and S27). As an inherent feature of its topology, the trefoil knot is chiral. However, the X‐ray diffraction analysis revealed the co‐crystallization of two mirror‐symmetric enantiomers in a 1:1 ratio in the centrosymmetric space group *P*–1 (Figure 2c).

The stability of [**3‐TK**](CF_3_SO_3_)_6_ in solution was further investigated. To probe the propensity of dissociation into monomeric metallamacrocycles, the concentration of [**3‐TK**](CF_3_SO_3_)_6_ in methanol was decreased from 13.0 to 2.0 mM. Upon lowering the concentration, the ^1^H NMR spectra remained unchanged, with no new signals appearing (Figure S22), indicating that the cation [**3‐TK**]^6+^ is remarkably stable in polar solvents and its integrity is independent of the concentration. Subsequently, the solvent polarity was decreased by preparing a chloroform solution at the low concentration of 2.0 mM. Again, the ^1^H NMR spectrum showed no new signals (Figure S23), confirming that [**3‐TK**]^6+^ is also highly robust in low‐polarity environments.

A closer inspection of the molecular structure of [**3‐TK**]^6+^ revealed two distinct π⋅⋅⋅π stacking modes between the dinuclear metal complexes **P^3^** and the biphenyl units of **L**. In mode one (observed twice), the naphthalene group of **P^3^** is stacked in an asymmetric fashion over a single phenyl ring of the biphenyl unit (Figure S40). In mode two, the naphthalene group is stacked in a symmetric fashion over the midpoint of the central biphenyl C_ph_‒C_ph_ bond. These different geometrical arrangements should in principle create distinctly different chemical environments for the biphenyl protons. Intriguingly, the solution‐state ^1^H NMR and ^1^H‐^1^H COSY spectra display only two symmetric doublets for the biphenyl protons, suggesting an averaged symmetric environment (Figures 2a, S15, and S18). This is further supported by the ^1^H‐^13^C HSQC spectrum, which shows that the biphenyl protons (H11 and H12) couple to only one set of biphenyl carbon atoms (Figure S20). These observations strongly suggest that in solution, the naphthalene and biphenyl units are not static but are dynamically sliding relative to each other. This motion averages the different stacking arrangements into a single, symmetric environment on the NMR timescale. Importantly, this internal sliding motion does not compromise the overall trefoil knot topology but rather imparts a degree of vibrational flexibility. We speculate that such well‐defined molecular vibrations could potentially be harnessed to generate photothermal effects and ultrasonic waves, opening up exciting avenues for applications in molecular acoustics. Unlike the well‐established shuttling of separate components in rotaxanes,^[^ ^55^ ^]^ or guest movements within a host cavity,^[^ ^56^ ^]^ our system demonstrates a distinct type of internal dynamics, where integral parts of a single, topological complex molecule slide against each other.

The significant red‐shift in the absorption of [**3‐TK**](CF_3_SO_3_)_6_, attributed to its unique trefoil knot topology and π⋅⋅⋅π stacking interactions, strongly suggested its potential as a photothermal agent. To evaluate this potential, aqueous solutions of [**3‐TK**](CF_3_SO_3_)_6_ with different concentrations (100, 300, 500, 700, and 900 µM, with 1% DMSO as a co‐solvent) were prepared in polypropylene tubes and irradiated with a 730 nm continuous‐wave laser. The temperature increase of each solution was monitored and recorded by a thermal imager (FLIR A300). First, the concentration‐dependent photothermal effect was investigated. As depicted in Figure 3b,c, the temperature elevation of the [**3‐TK**](CF_3_SO_3_)_6_ solutions was positively correlated with their concentration under a fixed laser power density (1.0 W cm^−^^2^). Notably, at a concentration of 900 µM, the solution temperature rapidly increased from ≈25° to 80 °C within 15 min, representing a remarkable temperature increment (Δ*T*) of 55 °C. In stark contrast, a control sample of pure water (containing 1% DMSO) exhibited a negligible temperature rise of only 2 °C under identical irradiation conditions (Figure S29). Furthermore, a clear power‐dependent heating effect was observed for a 300 µM solution of [**3‐TK**](CF_3_SO_3_)_6_, where higher laser power densities led to faster and greater temperature increases (Figure S30). Beyond efficacy, operational stability is a critical parameter. The photothermal stability of [**3‐TK**](CF_3_SO_3_)_6_ was therefore evaluated by subjecting a 300 µM solution to four consecutive laser‐on/off cycles. As shown in Figure 3d, the maximum temperature reached in each cycle showed no discernible decay, confirming the excellent photostability and operational robustness of [**3‐TK**](CF_3_SO_3_)_6_ as a photothermal transducer.

To elucidate the contribution of the unique trefoil knot topology to the photothermal performance, we performed a comparative experiment with [**3‐TK**](CF_3_SO_3_)_6_ and dinuclear building block **P^3^**. Since each molecule of [**3‐TK**](CF_3_SO_3_)_6_ is assembled from three units of **P^3^**, solutions with stoichiometrically equivalent chromophore concentrations were compared. A 300 µM solution of [**3‐TK**](CF_3_SO_3_)_6_ reached a peak temperature of 55 °C, significantly outperforming the 900 µM solution of **P^3^**, which only reached 34 °C under the same conditions (Figure 3e). The weak photothermal response observed for **P^3^**, in contrast to [**3‐TK**](CF_3_SO_3_)_6_, must be attributed to its significantly weaker absorption at 730 nm (Figure S27). The excellent photothermal performance of [**3‐TK**](CF_3_SO_3_)_6_ was further confirmed when comparing a less concentrated 100 µM solution of [**3‐TK**](CF_3_SO_3_)_6_ with a 300 µM **P^3^** solution, where the trefoil knot again demonstrated substantially higher heating capability (Figure S31). Contrary to this, the tetranuclear macrocycle [**2**](CF_3_SO_3_)_4_, also featuring a half‐sandwich metal complex, showed a negligible temperature increase of only ≈3 °C (Figure S32). These findings clearly demonstrate that the formation of the hexanuclear trefoil knot architecture, with its enhanced π⋅⋅⋅π stacking and structural integrity, synergistically amplifies the photothermal conversion process, rather than it being a simple additive effect of its constituent units.

To quantify the photothermal performance, the photothermal conversion efficiency (*η*) of [**3‐TK**](CF_3_SO_3_)_6_ was calculated based on its time‐dependent temperature profile. A 300 µM aqueous solution was irradiated with a 730 nm laser (1.0 W cm^−^^2^, 0.34 cm^2^ spot radius) until thermal equilibrium was reached. The subsequent temperature decay curve upon switching off the laser was recorded (Figure S29). The absorbance of a 300 µM aqueous solution of [**3‐TK**](CF_3_SO_3_)_6_ at 730 nm is shown in Figure S28. For the calculation, we analyzed the data from a comprehensive 500 s cooling interval to ensure the model reflects a realistic and stable heat dissipation process. From the linear fit of this data (Figure S33), the crucial time constant of heat transfer (*T_s_*) was determined to be 335.4 s. Based on these parameters and the detailed method outlined in the Supporting Information (Section S5), [**3‐TK**](CF_3_SO_3_)_6_ was found to possess an exceptionally high photothermal conversion efficiency of 73.5%. This value represents a substantial improvement over the ≈60% efficiency typically achieved by many reported supramolecular assemblies that rely solely on mechanisms like π···π stacking.^[^ ^57^, ^58^ ^]^ The superiority of [**3‐TK**](CF_3_SO_3_)_6_ is attributed to a powerful synergy where the unique half‐sandwich building block works in concert with π···π stacking to induce profound fluorescence quenching, thereby maximizing the channeling of absorbed energy into non‐radiative heat generation.

The trefoil knot exhibits a remarkable efficiency to channel absorbed photon energy into non‐radiative decay pathways, which is based on the enhanced molecular vibrations within the knotted core. While this process is highly effective at generating heat for photothermal applications, the rapid and localized thermal effect resulting from these vibrations is also the fundamental principle of the PA effect. This dual potential prompted our subsequent investigations into the PA properties of [**3‐TK**](CF_3_SO_3_)_6_.

**Figure 4:**
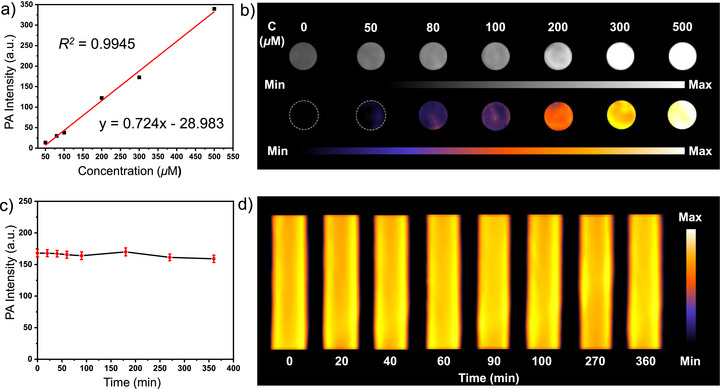
a) PA signal intensities and b) PA images of aqueous solutions of [**3**‐TK](CF_3_SO_3_)_6_ (300 µM, with 1% DMSO) at different concentrations under 730 nm laser photoirradiation. c) Long‐term PA signal intensities and d) PA images of aqueous solutions of [**3**‐TK](CF_3_SO_3_)_6_ (300 µM, with 1% DMSO) under 730 nm photoirradiation.

Aqueous solutions of [**3‐TK**](CF_3_SO_3_)_6_ with different concentrations (50, 80, 100, 300, and 500 µM, containing 1% DMSO) were irradiated with a 730 nm pulsed laser in order to generate ultrasound signals. As illustrated in the PA images (Figure 4b), a distinct‐concentration dependent brightening was observed. The PA signal became progressively more intense as the concentration of [**3‐TK**](CF_3_SO_3_)_6_ increased. Quantitative analysis further confirmed this trend, revealing a strong linear correlation between the PA signal intensity and the [**3‐TK**](CF_3_SO_3_)_6_ concentration (Figure 4a).

Next, we assessed the long‐term photostability, a critical feature for any practical PA contrast agent. Remarkably, after continuous laser irradiation for 6 h, the PA signal of an aqueous solution of [**3‐TK**](CF_3_SO_3_)_6_ (300 µM) exhibited no significant degradation (Figure 4c,d). This result highlights the exceptional photostability of [**3‐TK**](CF_3_SO_3_)_6_, ensuring reliable and consistent signal generation over extended periods.

Finally, in order to unequivocally confirm that the observed PA signal originates from the intrinsic optical properties of the trefoil knot, we measured its PA spectrum. As depicted in Figures S34 and S35, the spectrum, which plots PA signal intensity as a function of excitation wavelength, showed excellent congruence with the UV–vis‐NIR absorption spectrum of [**3‐TK**](CF_3_SO_3_)_6_ (Figure S27). This strong correlation provides definitive evidence that the observed PA effect is directly driven by the light absorption characteristics inherent to the unique architecture of [**3‐TK**](CF_3_SO_3_)_6_.

## Conclusion

We present a self‐assembly strategy based on the size of the building blocks and thus enabling topological control in the construction of a [3+3] metallosupramolecular trefoil knot. A key property of this trefoil knot is its notable structural robustness in solution. This stability proved crucial, permitting a detailed investigation of its photophysical properties. Structural studies established that the knot's topology enforces strong intramolecular π⋅⋅⋅π stacking interactions, which in turn results in near‐infrared absorption. This optical feature leads to efficient photothermal conversion and a remarkable PA response. Thus, we demonstrated that appropriately stabilized metallosupramolecular knots can function as effective PA agents, highlighting a promising, yet largely unexplored, application for these architectures. The design principles established here may provide a foundation for the rational synthesis of other intricate topologies with tailored functions.

## Conflict of Interests

The authors declare no conflict of interest.

## Supporting information



Supplementary Information

Supplementary Information

## Data Availability

The data that support the findings of this study are available in the Supporting Information of this article.
