# Supplementary material for: Observation of Near‐Infrared Photothermal and Photoacoustic Effects in a Metallosupramolecular Trefoil Knot
Source: Angew Chem Int Ed Engl. 2025 Oct 3;64(48):e202518415. doi: 10.1002/anie.202518415 (PMC12643334; doi:10.1002/anie.202518415)
Supplement: Supplementary file 1 — Supplementary Information [file ANIE-64-e202518415-s002.pdf]

Table of Contents

1. Experimental procedures……………………………………………..S2
2. NMR and ESI-MS spectra………………………………………………S4
3. Ultraviolet-visible absorption spectra………………………………. S17
4. Photothermal performance……………………………………………S19
5. Calculation of the photothermal conversion efficiency………………..S22
6. Photoacoustic performance…………………………………………...S23
7. X-ray diffraction studies………………………………………………..S24
8. References ……………………………………………………………..S31

1. Experimental procedures

General Procedures. All manipulations were carried out under an argon atmosphere unless stated otherwise. ^{1}H and $^{13}C\{^{1}H\}$ NMR spectra were measured on a Bruker ASCEND-TM 400 spectrometer at ambient temperature (295 K). Chemical shifts (δ) are expressed in ppm relative to $SiMe_4$ using the residual protonated solvent signal as an internal standard. For the assignments of the NMR resonances see the numbering at the molecular plots. Coupling constants are expressed in Hz. Mass spectra were obtained with reflex an Orbitrap LTQ XL spectrometer (Thermo Scientific). Absorption spectra were recorded on Lambd650 UV and visible spectrophotometer. Photothermal conversion experiments were performed under 730 nm laser beam irradiation (Changchun New Industries optoelectronics Tech Co., Ltd. China) and the temperatures were recorded with an IR thermal camera (FLIR A310 infrared camera). Photoacoustic experiments were performed under a 3D optoacoustic imaging system (LOIS-3D, TomoWave Suzhou Medical Imaging Co., Ltd, China). The binuclar nickel(**II**) NHC precursor **P^{1}**[S1] and bis(imidazole) ligand **L** (L = 4,4'-bis((1*H*-imidazol-1-yl)methyl)-1,1'-biphenyl)[S2] were prepared as previously described.

Preparation of complex [1](OTf)$_2$. To a solution of precursor **P^{1}** (32.88 mg, 0.04 mmol) in dichloromethane (10 mL) was added bis(imidazole) ligand **L** (12.58 mg, 0.04 mmol) and AgOTf (20.56 mg, 0.08 mmol) suspended in methanol (10 mL). The suspension was stirred at ambient temperature for 12 h. Subsequently, insoluble compounds (AgI) were removed by filtration. The filtrate was concentrated to a volume of 5 mL under reduced pressure. Slow diffusion of diethyl ether into the concentrated filtrate yielded complex [**1**](OTf)$_2$ as a green powder. Yield: 44.77 mg (0.038 mmol, 95%). ^{1}H NMR (400 MHz, CD_3OD): δ = 8.45 (d, *J* = 9.2 Hz, 2H, H4), 8.08 (d, *J* = 9.2 Hz, 2H, H3), 7.32 (s, 2H, H10), 7.20 (s, 2H, H9), 7.07 (s, 2H, H11), 6.78 (d, *J* = 8.3 Hz, 4H, H14), 6.71 (d, *J* = 8.3 Hz, 4H, H15), 5.81 (m, 2H, H6’), 5.70 (m, 2H, H6’), 5.66 (s, 10H, H8), 5.57 (m, 2H, H6), 5.20 (m, 2H, H6), 5.11 (d, 2H, H12), 5.10 (d, 2H, H12’), 1.53 (t, *J* = 7.2 Hz,

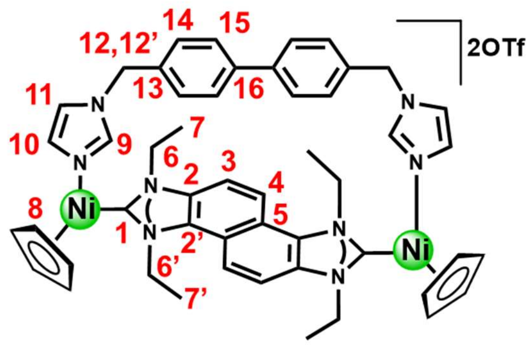

6H, H7), 1.28 (t, J = 7.2 Hz, 6H, H7'). $^{13}C\{^1H\}$NMR (100 MHz, CD_3OD): δ = 174.90 (C1), 141.52 (C9), 141.21 (C13), 134.80 (C16), 134.59 (C11), 132.23 (C2), 129.75 (C2'), 127.59 (C14), 126.61 (C15), 122.65 (C10), 118.01 (C4), 117.25 (C5), 111.77 (C3), 92.49 (C8), 50.62 (C12, C12'), 47.70 (C6'), 44.88 (C6), 14.46 (C7'), 14.38 (C7). HRMS (ESI, positive ions): m/z =1029.2544 (calcd for [**1**+OTf]$^+$ 1029.2537), 440.1591(calcd for [**1**]$^{2+}$ 440.1505).

Preparation of complex [2](OTf)$_4$. A methanol solution of $[Cp^*RhCl_2]_2$ (24.72 mg, 0.04 mmol) was added to a solution of 2-(1H-benzimidazol-2-yl)-1H-benzimidazole (9.37mg, 0.04 mmol) and NaOH (3.20 mg, 0.08 mmol) in methanol (40 mL). The suspension was stirred at ambient temperature for 6 h. Then AgOTf (41.11mg, 0.16 mmol) was added to the mixture which was stirred for another 3 h, followed by filtration to remove insoluble compounds (AgCl and NaCl). A methanol solution (about 10 mL) of bis(imidazole) ligand **L** (12.58 mg, 0.04 mmol) was then added to the filtrate. After the solution was stirred at ambient temperature for 12 h The reaction mixture was concentrated to a volume of 8 mL under reduced pressure. Filtration through Celite and slow diffusion of diethyl ether into the filtrate (including several drops of DMSO) yielded an orange crystalline solid. Yield 48.65 mg (0.018 mmol, 90%). ^1H NMR (400 MHz, CD_3OD/DMSO-d_6, 4:1, v/v): δ = 8.00 (m, 8H, H3), 7.85 (s, 4H, H7), 7.54 (m, 8H, H4), 7.20 (d, J = 8.4 Hz, 8H, H12), 6.75 (d, J = 8.4 Hz, 8H, H13), 6.00 (s, 4H, H8), 5.33 (s, 4H, H9), 4.72 (s, 8H, H10), 1.97 (s, 60H, H6). $^{13}C\{^1H\}$NMR (100 MHz, CD_3OD/DMSO-d_6, 4:1, v/v): δ = 156.69 (C1), 144.38 (C2), 139.58 (C11), 138.80 (C7), 135.16 (C14), 128.14 (C13), 128.09 (C9), 126.99 (C12), 122.93 (C4), 120.04 (C8), 119.19 (OTf), 116.06 (C3), 96.71 (C5), 50.05 (C10), 9.34 (C6). HRMS (ESI, positive ions): m/z = 1171.2257 (calcd for [**2**+2OTf]$^{2+}$ 1171.2252), 731.4446 (calcd for [**2**+OTf]$^{3+}$ 731.5003).

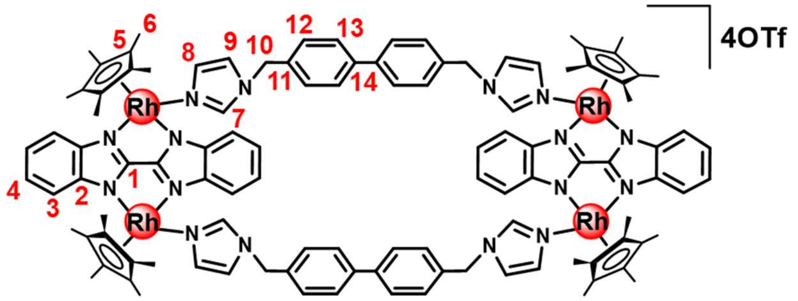

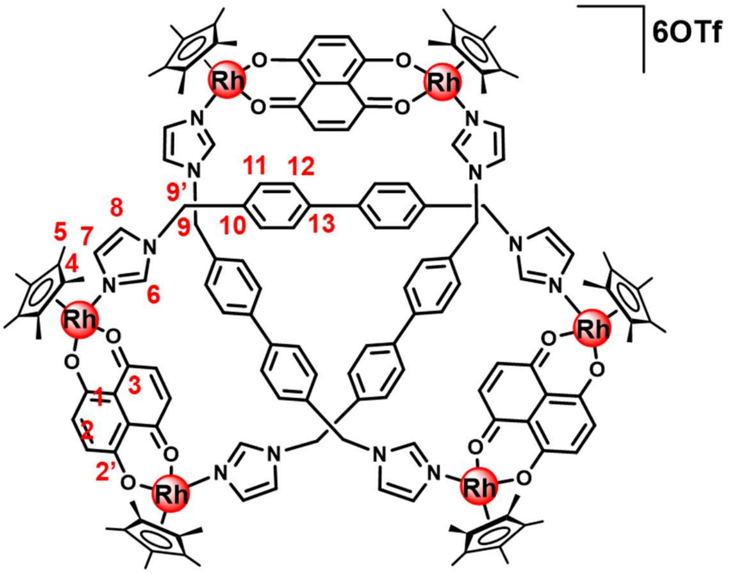

Preparation of complex [3-TK](OTf)$_6$. A methanol solution of $[Cp^*RhCl_2]_2$ (24.72 mg, 0.04 mmol) was added to a solution of 5,8-dihydroxy-1,4-naphthoquinone (7.61 mg, 0.04 mmol) and NaOH (3.20 mg, 0.08 mmol) in methanol (40 mL). The suspension was stirred at ambient temperature for 6 h. Subsequently, AgOTf (41.11 mg, 0.16 mmol) was added and the mixture was stirred for an additional 3 h. Filtration to remove insoluble compounds (AgCl and NaCl) yielded a clear solution. To this was added a methanol solution (about 10 mL) of bis(imidazole) ligand **L** (12.58 mg, 0.04 mmol). The mixture was stirred at ambient temperature for 12 h and then concentrated a volume of 8 mL under reduced pressure. Filtration through Celite and slow diffusion of diethyl ether into the filtrate (including several drops of DMSO) yielded [**3-TK**](OTf)$_6$ as a green crystalline solid. Yield: 46.88 mg (0.0122 mmol, 92%). ^{1}H NMR (400 MHz, CD_3OH): δ = 8.39 (s, 6H, H6), 7.74 (s, 6H, H7), 6.90 (s, 6H, H8), 6.85 (d, J = 8.2 Hz, 12H, H11), 6.65 (d, J = 9.9 Hz, 6H, H2), 6.38 (*d*, J = 9.9 Hz, 6H, H2'), 5.28 (d, J = 14.0 Hz, 6H, H9), 5.03 (d, J = 14.0 Hz, 6H, H9'), 4.52 (d, J = 8.2 Hz, 12H, H12), 1.50 (*s*, 90H, H5). ^{13}C{^{1}H}NMR (100 MHz, CD_3OH): δ = 171.20 (C3), 170.85 (C1), 138.16 (C2), 138.13 (C13), 137.22 (C7), 136.64 (C2'), 133.34 (C10), 128.20 (C11), 128.04 (C8), 126.33 (C12), 121.54 (C6), 119.17 (OTf), 93.83 (C4), 51.14 (C9, C9'), 6.78 (C5). HRMS (ESI, positive ions): m/z = 1766.4950 (calcd for [**3-TK**+4OTf]$^{2+}$1766.2208), 808.4334(calcd for [**3-TK**+2OTf]$^{4+}$ 808.3836).

2. NMR and ESI-MS spectra

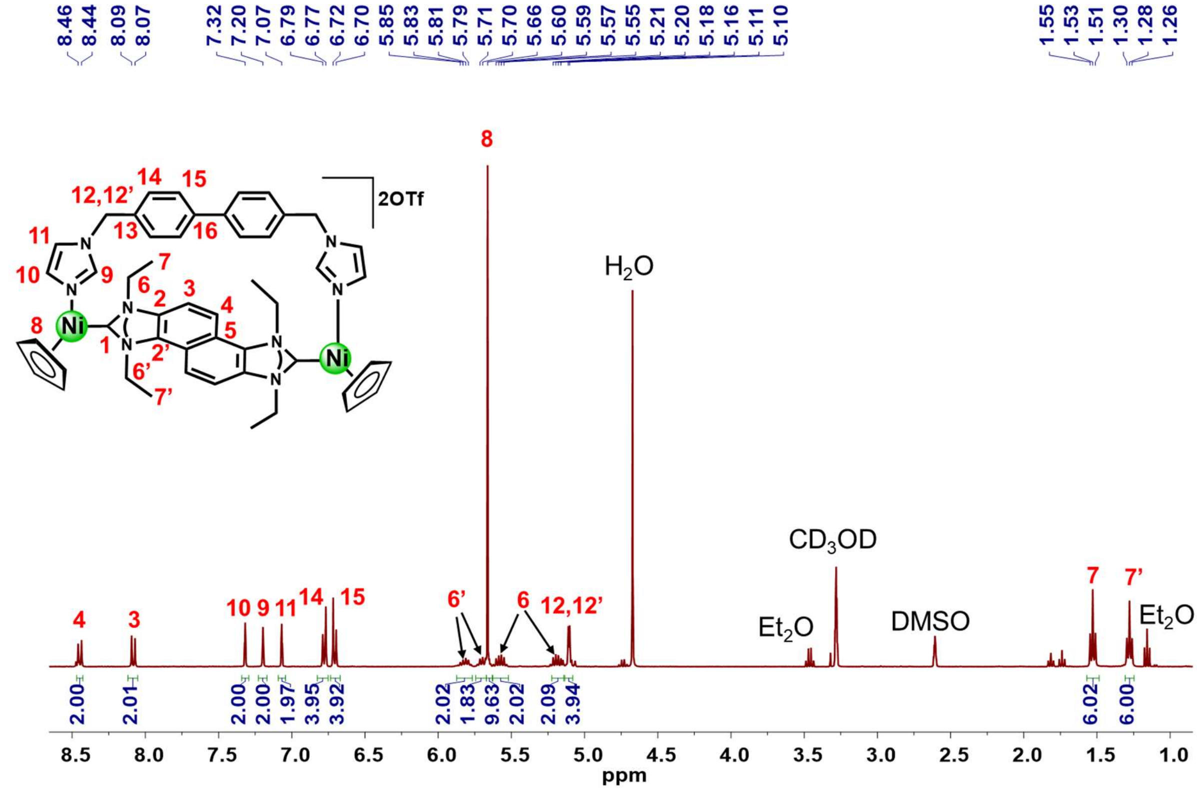

Figure S1. ^{1}H NMR spectrum of [**1**](OTf)2 in CD3OD ([12.0 mM], 400 MHz).

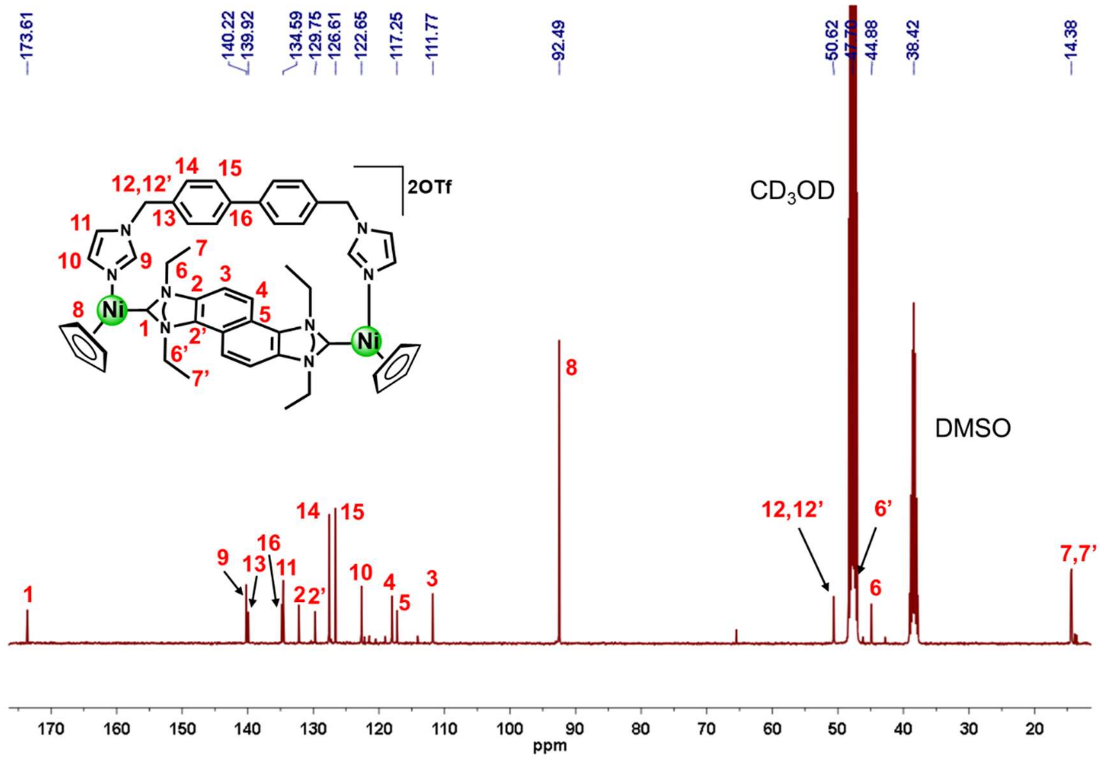

Figure S2. ^{13}C NMR spectrum of [**1**](OTf)2 in CD3OD ([12.0 mM], 100 MHz).

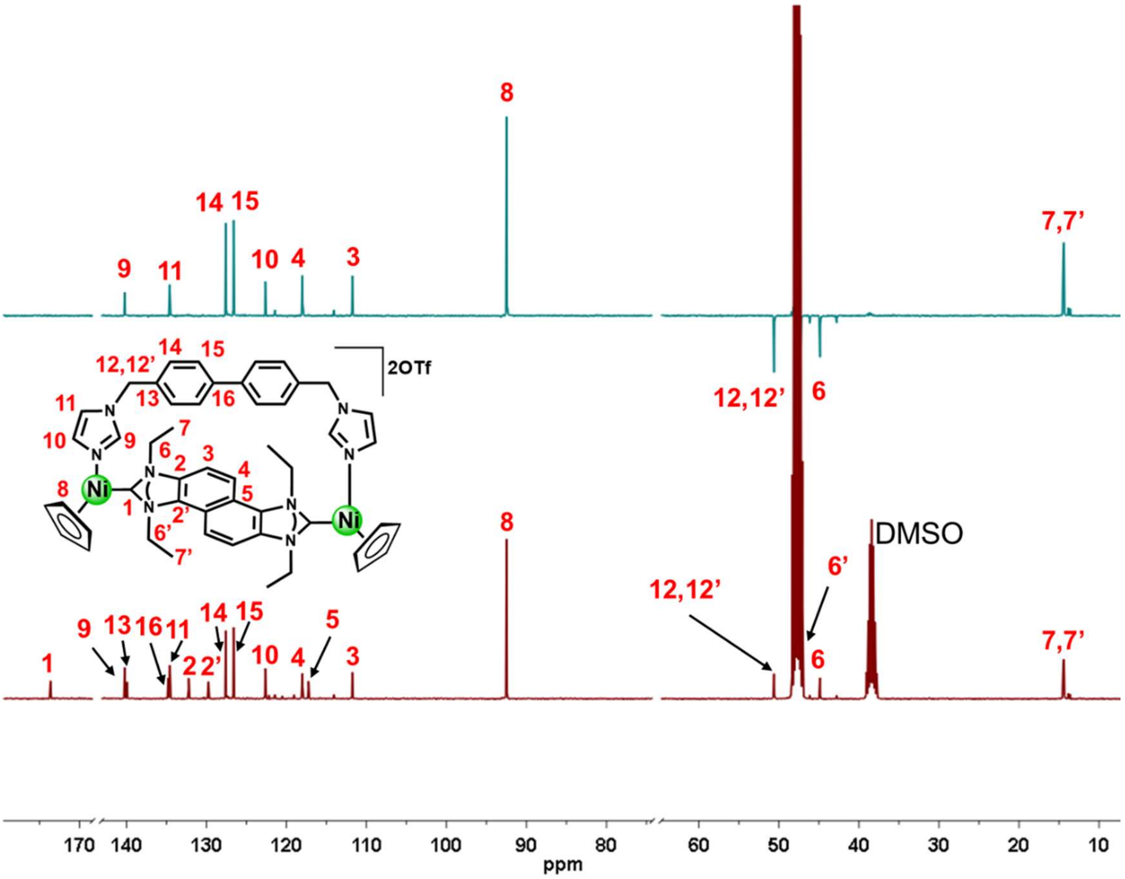

Figure S3. DEPT-135 (top) and ^{13}C NMR spectra (bottom) of [**1**](OTf)$_2$ in CD_3OD ([12.0 mM], 100 MHz).

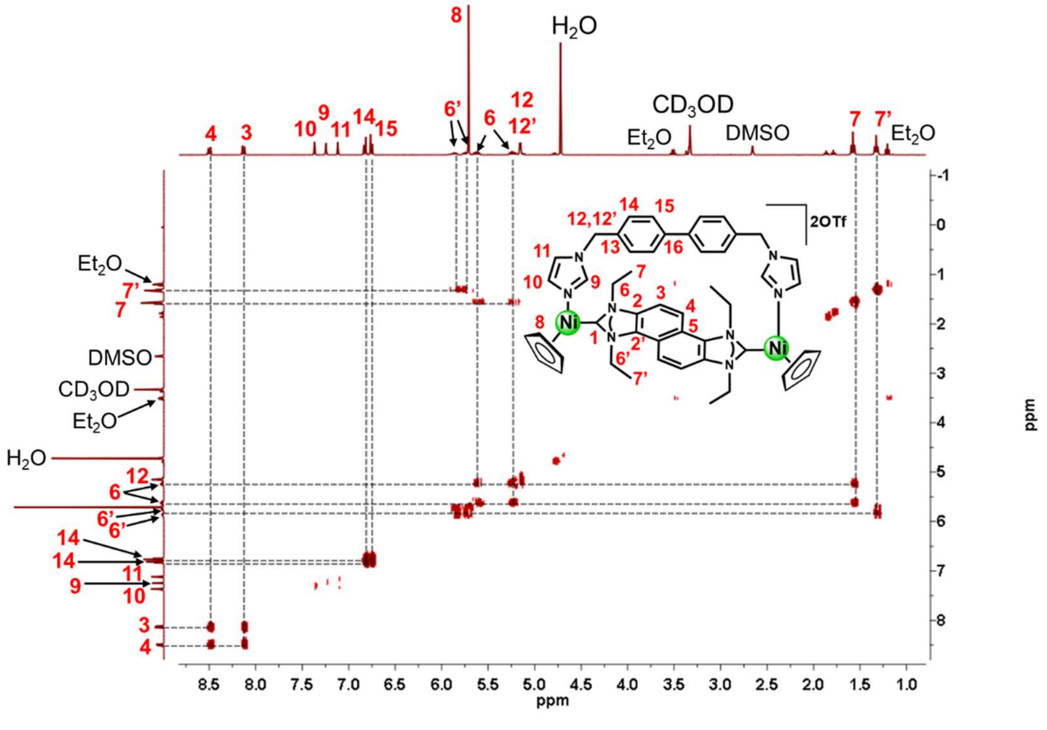

Figure S4. ^1H-^1H COSY NMR spectrum of [**1**](OTf)$_2$ in CD_3OD, ([12.0 mM], 100 MHz).

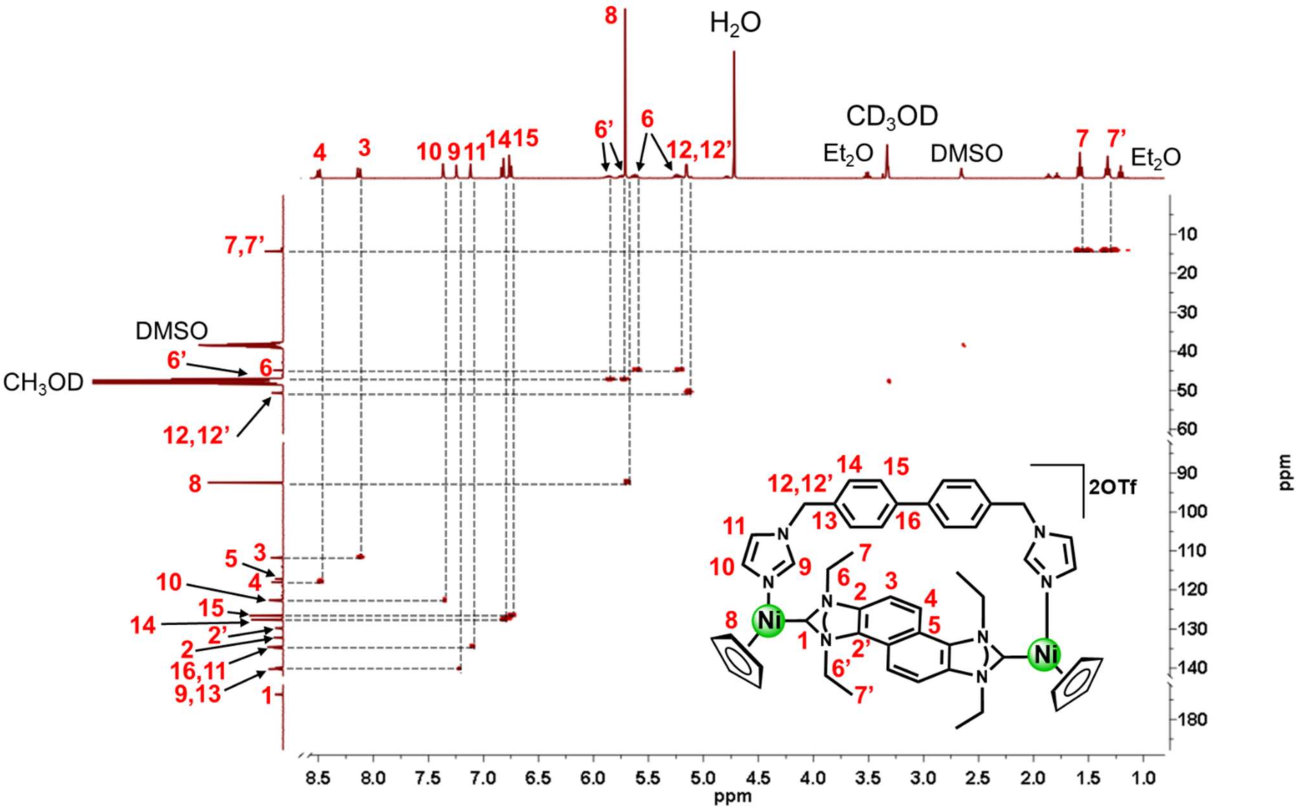

Figure S5. ^{1}H-^{13}C HSQC spectrum of [**1**](OTf)$_2$ in CD_3OD [12.0 mM].

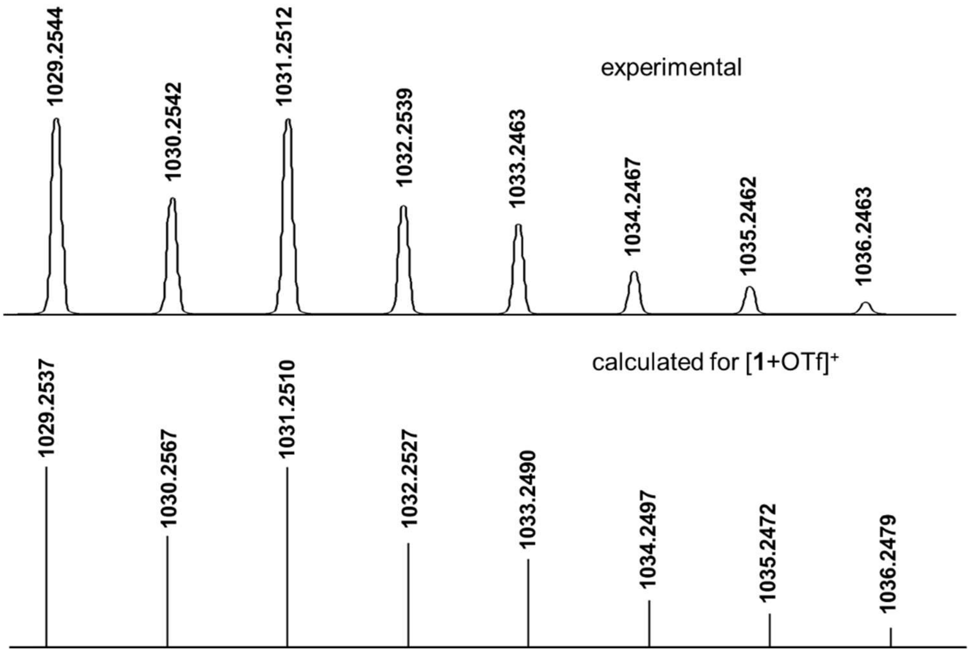

Figure S6. Section of the experimental (top) and calculated (bottom) ESI HRMS spectrum of [**1**](OTf)$_2$.

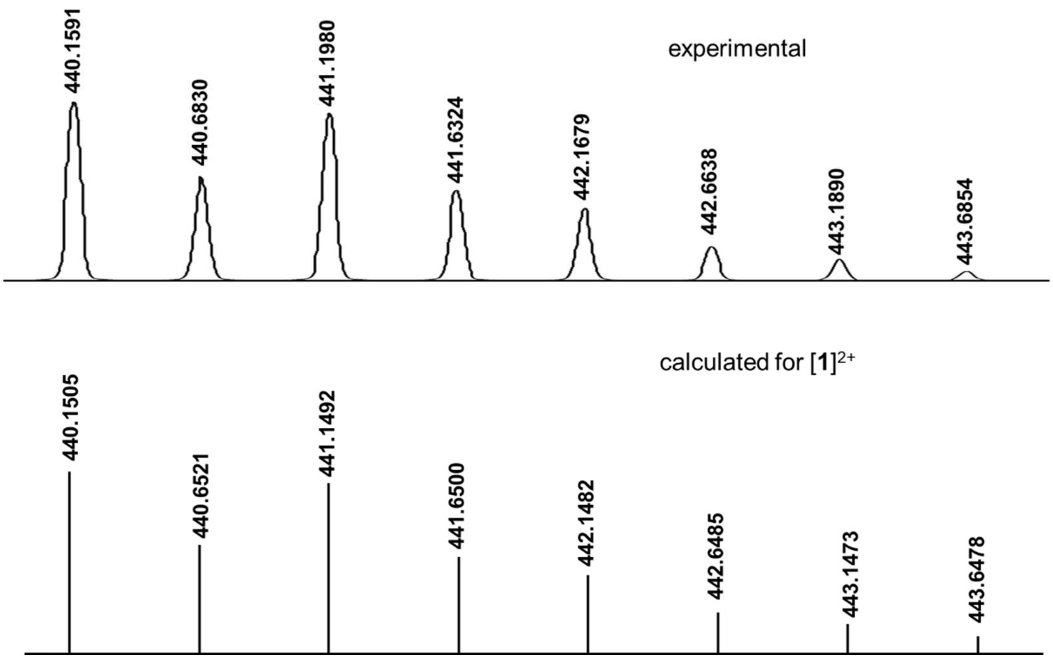

Figure S7. Section of the experimental (top) and calculated (bottom) ESI HRMS spectrum of [**1**]$(OTf)_2$.

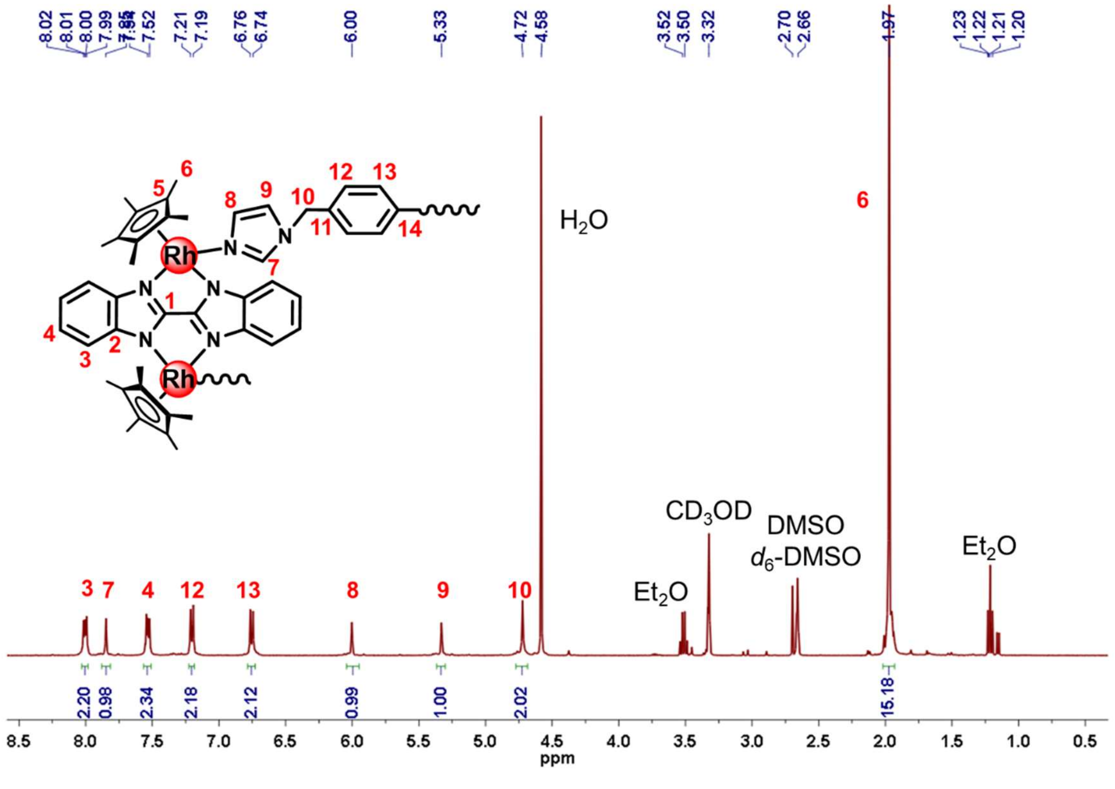

Figure S8. ^{1}H NMR spectrum of [**2**]$(OTf)_4$ in the solvent mixture CD_3OD/d_6-DMSO (CD_3OD: d_6-DMSO = 4:1, v/v), [12.0 mM], 400 MHz.

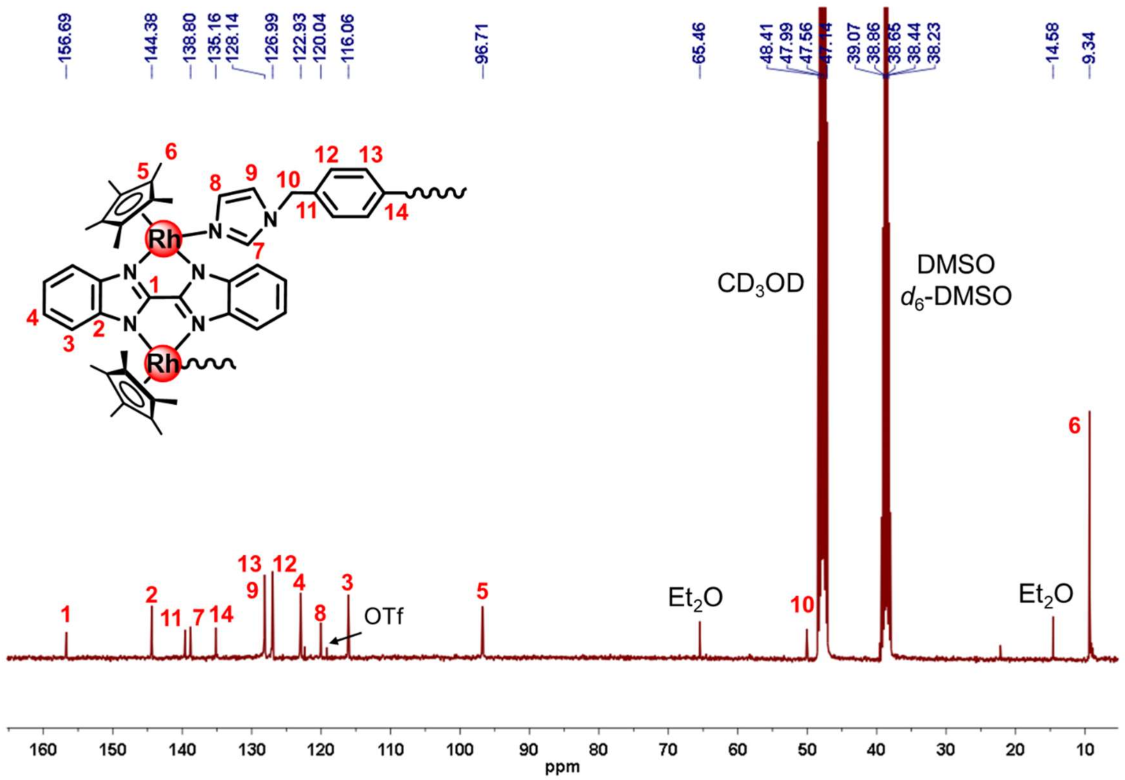

Figure S9. ^{13}C NMR spectrum of [**2**](OTf)$_4$ in the solvent mixture CD_3OD/*d*$_6$-DMSO (CD_3OD: *d*$_6$-DMSO = 4:1, v/v), [12.0 mM], 100 MHz.

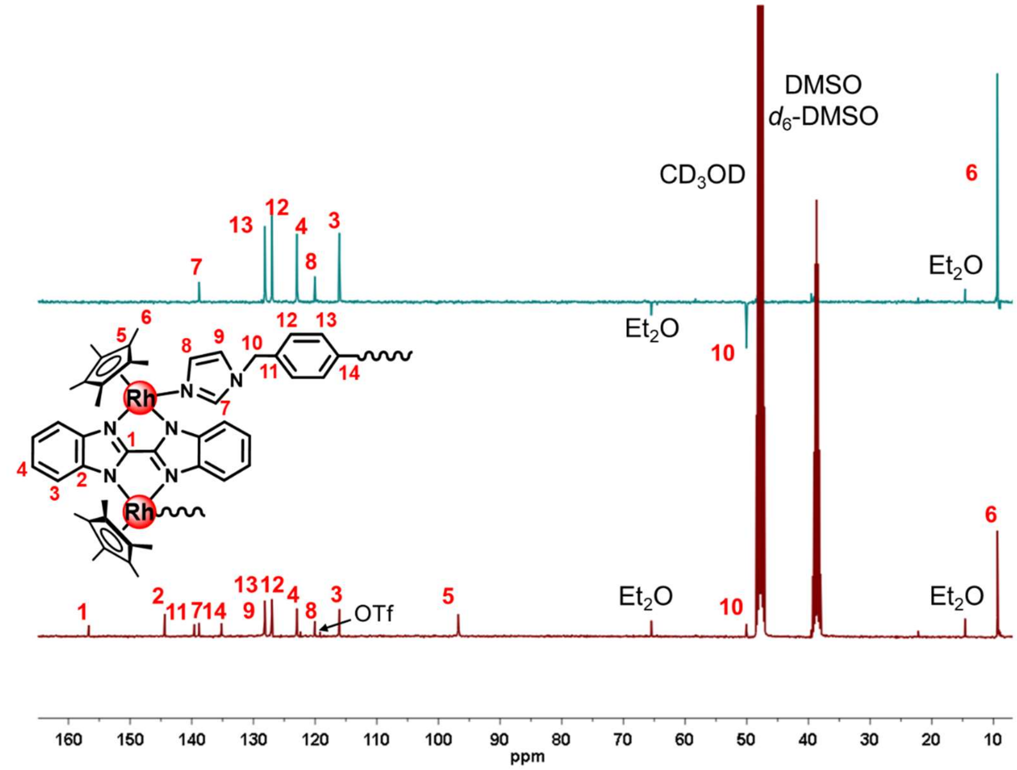

Figure S10. DEPT-135 (top) and ^{13}C NMR spectra (bottom) of [**2**](OTf)$_4$ in the solvent mixture CD_3OD/d$_6$-DMSO (CD_3OD:d$_6$-DMSO = 4:1, v/v), [12.0 mM], 100 MHz.

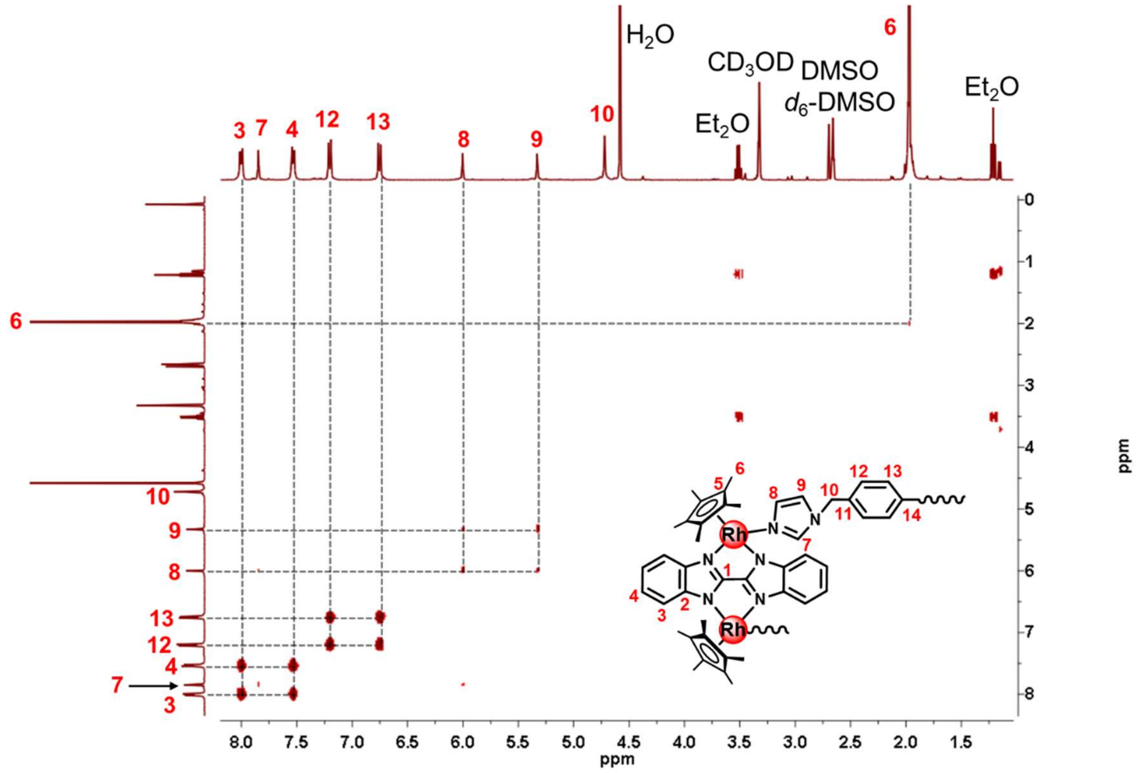

Figure S11. ^1H-^1H COSY NMR spectrum of [**2**](OTf)$_4$ in the solvent mixture CD_3OD/d_6-DMSO (CD_3OD:d_6-DMSO = 4:1, v/v), [12.0 mM], 100 MHz.

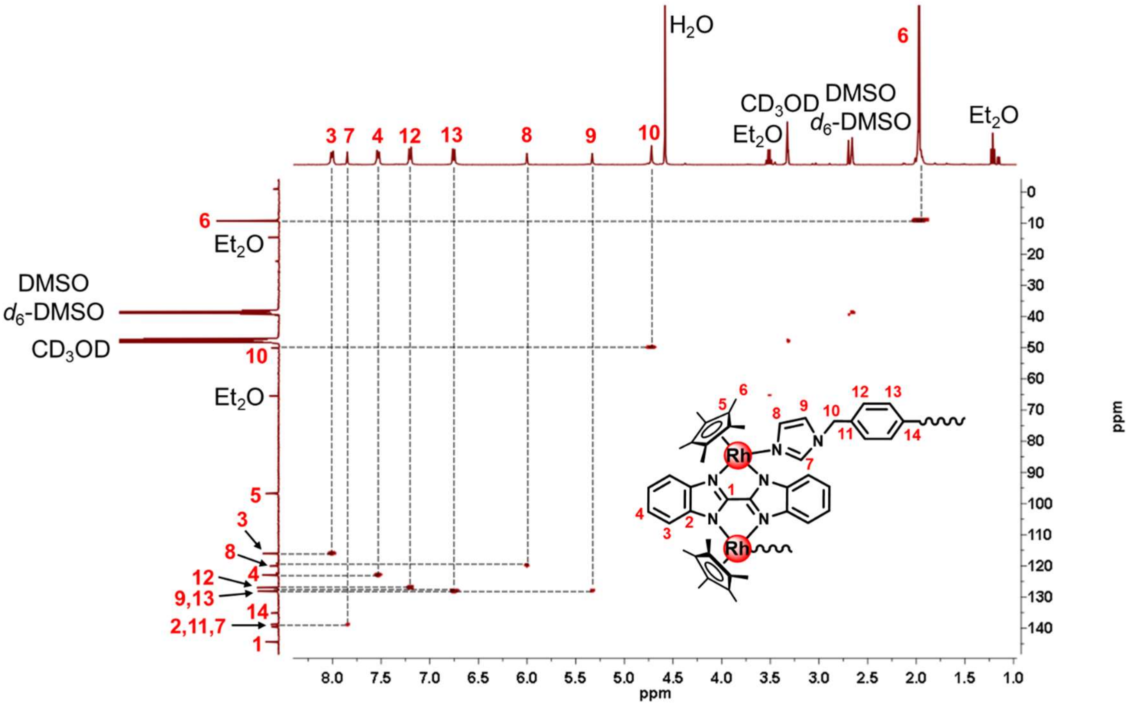

Figure S12. ^1H-^{13}C HSQC spectrum of [**2**](OTf)$_4$ in the solvent mixture CD_3OD/d_6-DMSO (CD_3OD: d_6-DMSO = 4:1, v/v), [12.0 mM].

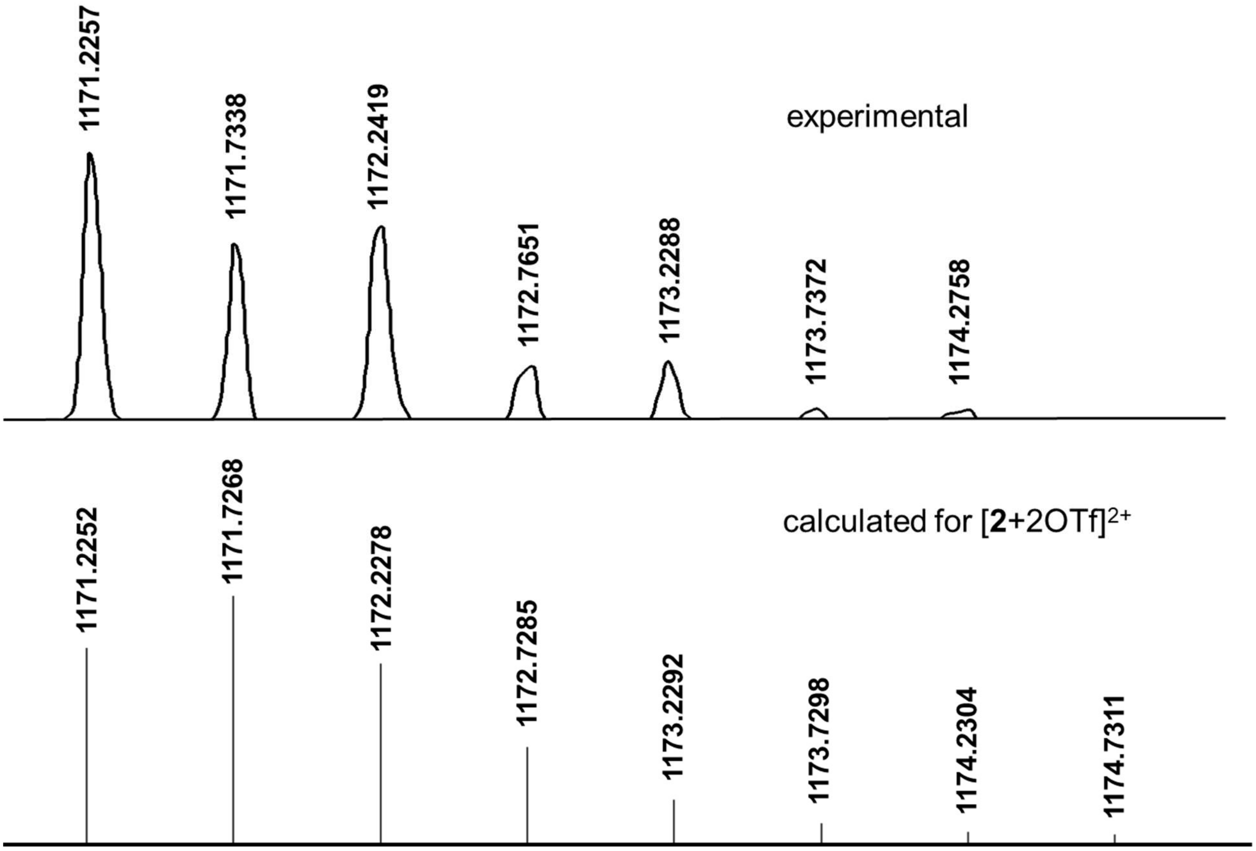

Figure S13. Section of the experimental (top) and calculated (bottom) ESI HRMS spectrum of [**2**]$(OTf)_4$.

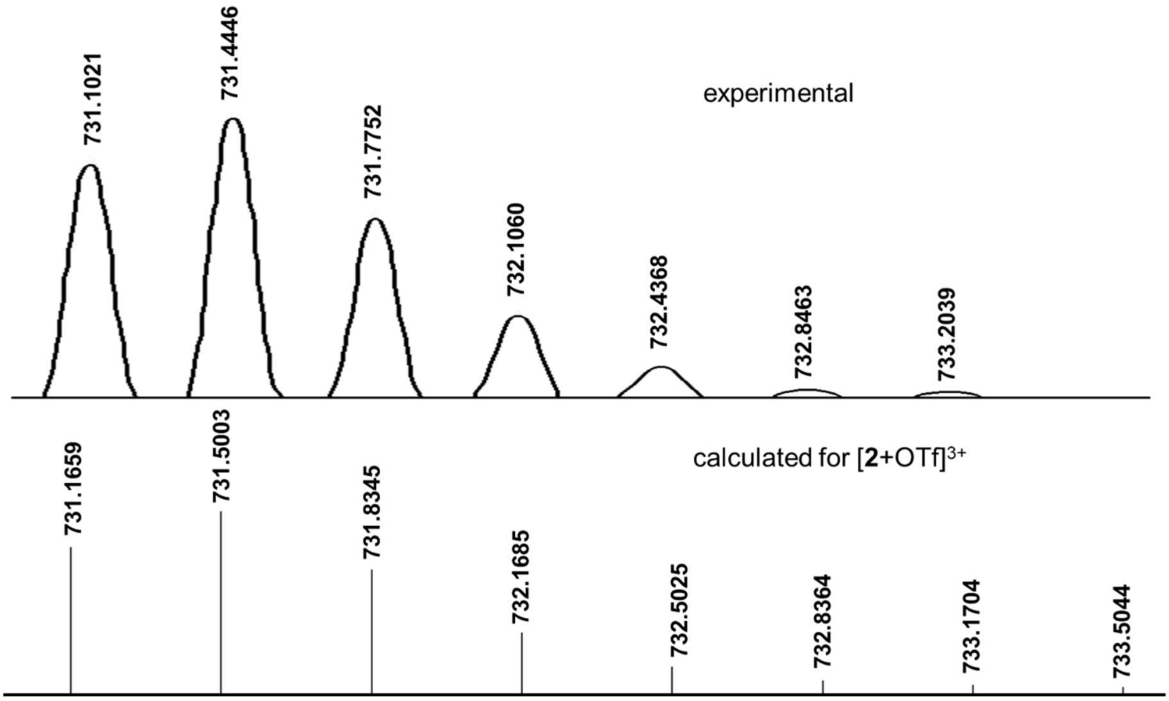

Figure S14. Section of the experimental (top) and calculated (bottom) ESI HRMS spectrum of [**2**]$(OTf)_4$.

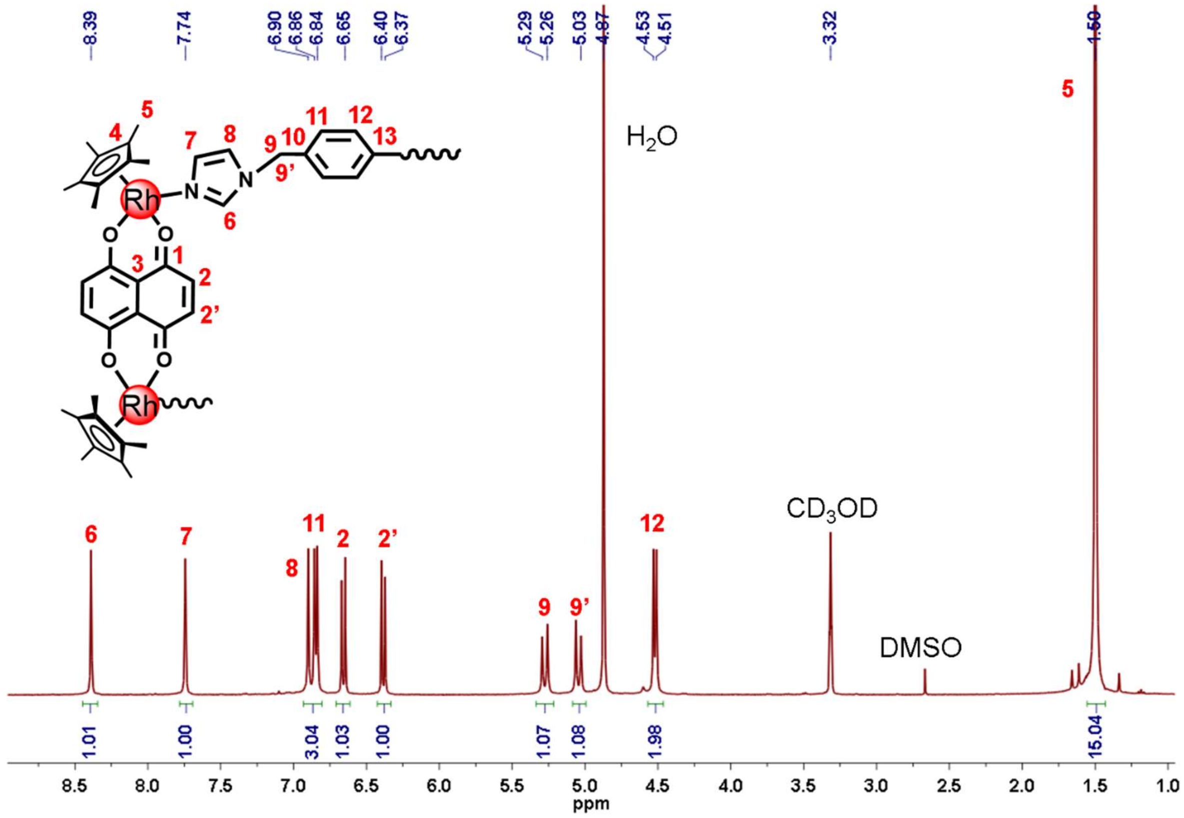

Figure S15. ^{1}H NMR spectrum of [**3-TK**]$(OTf)_6$ in CD_3OD, [13.0 mM], 400 MHz.

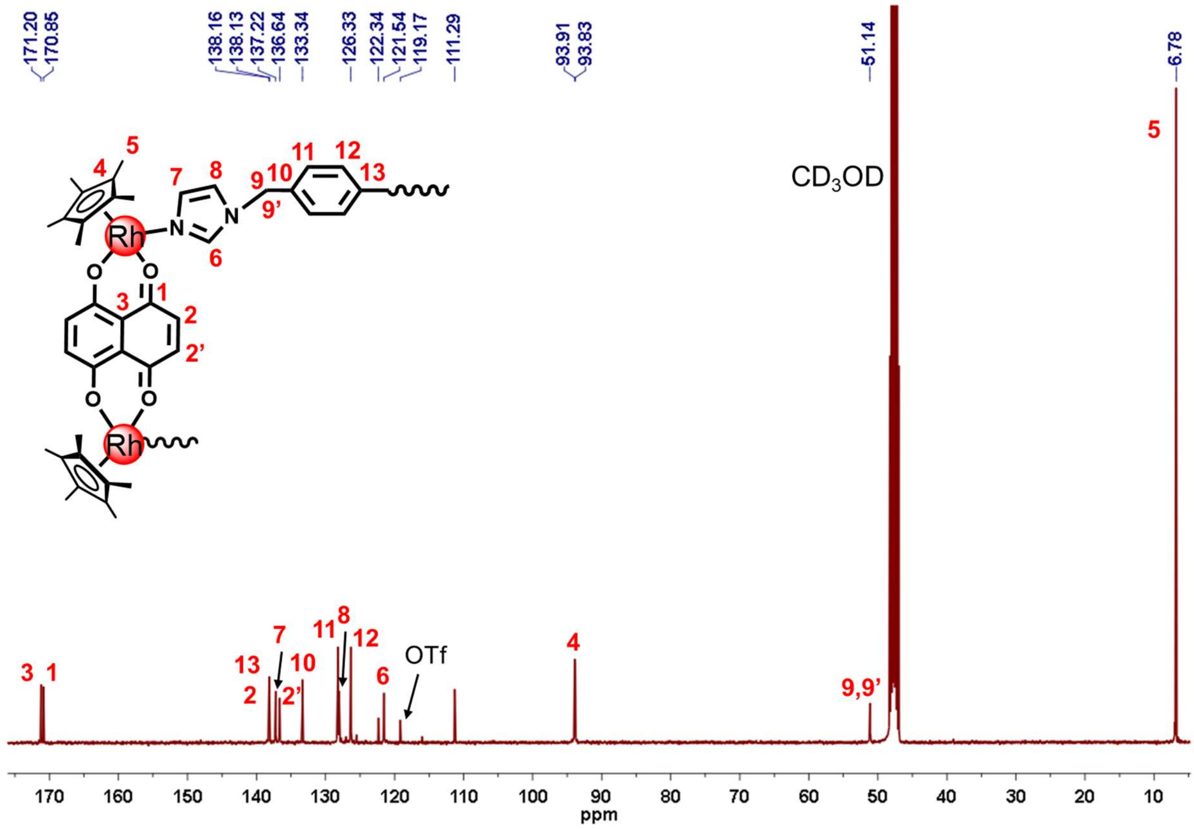

Figure S16. ^{13}C NMR spectrum of [**3-TK**]$(OTf)_6$ in CD_3OD, [13.0 mM], 100 MHz.

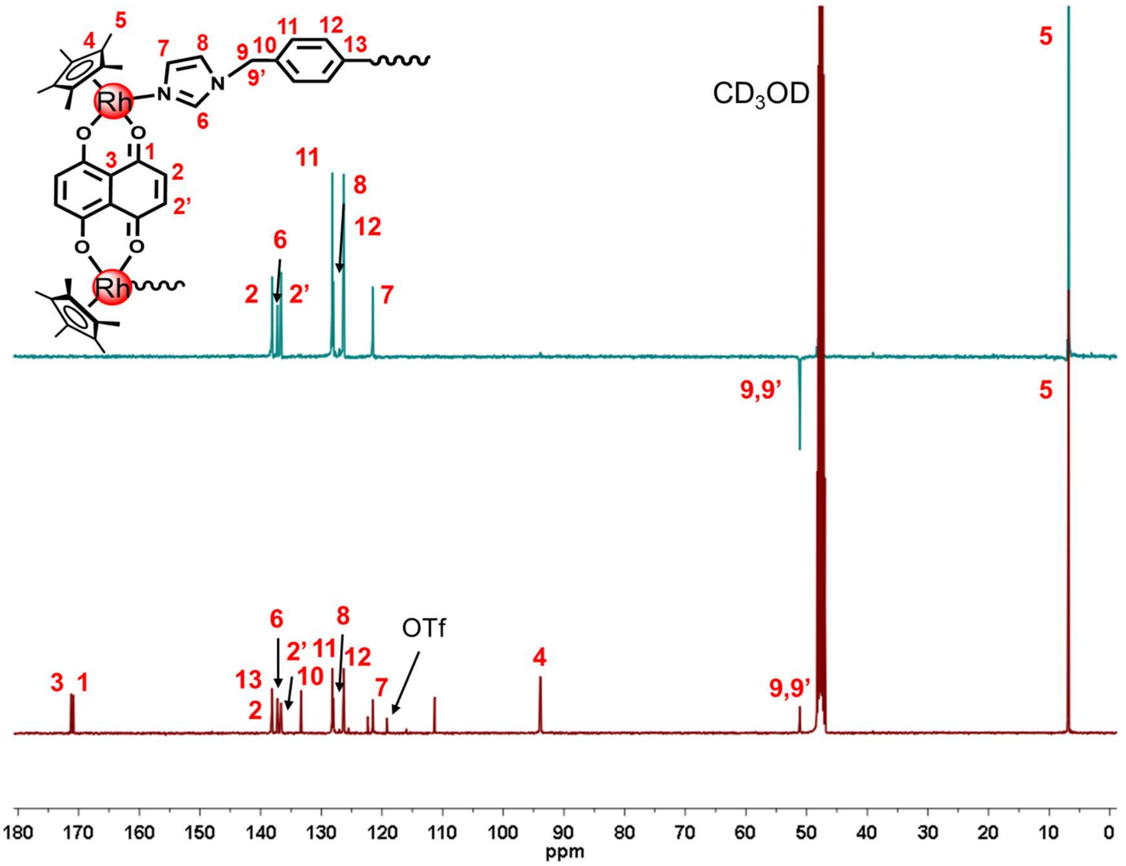

Figure S17. DEPT-135 (top) and ^{13}C NMR spectra (bottom) of [**3-TK**]$(OTf)_6$ in CD_3OD, [13.0 mM], 100 MHz.

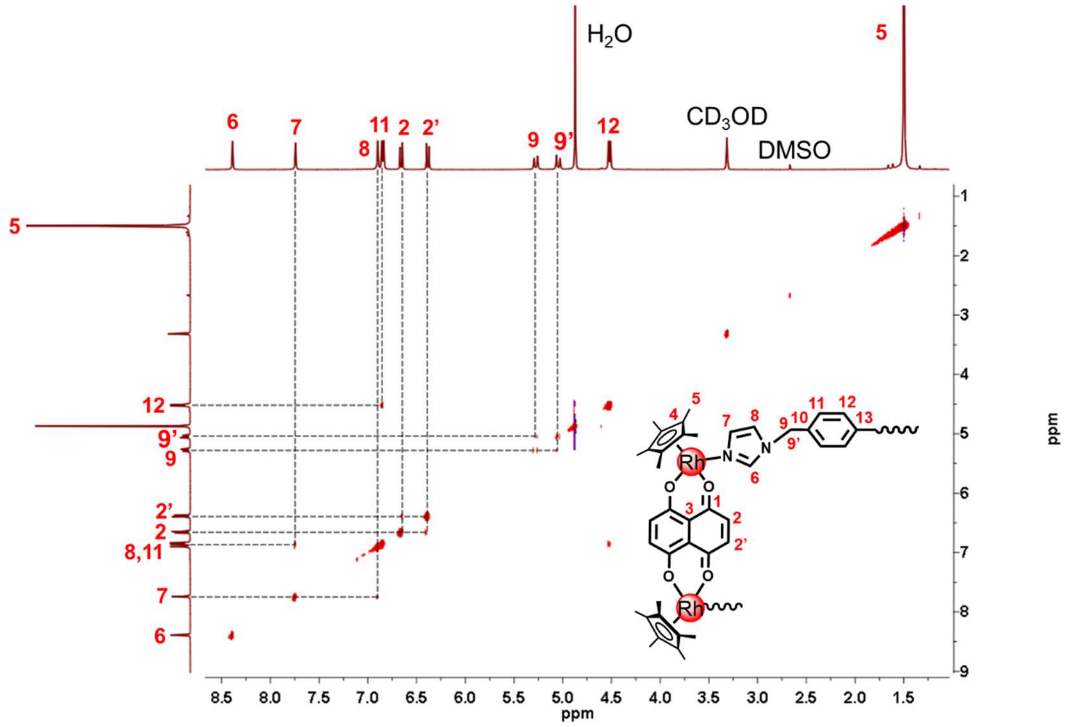

Figure S18. ^1H-^1H COSY NMR spectrum of [**3-TK**]$(OTf)_6$ in CD_3OD, [13.0 mM], 100 MHz.

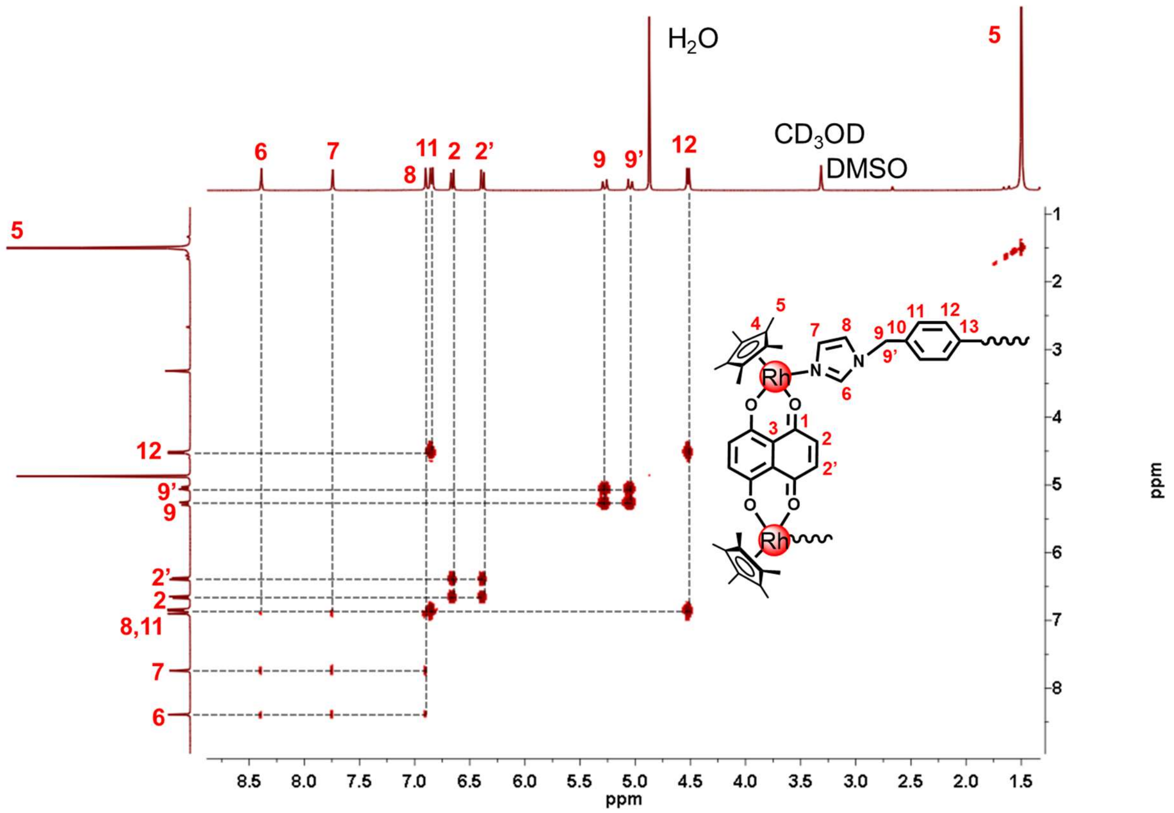

Figure S19. ^{1}H-^{1}H NOESY NMR spectrum of [**3-TK**]$(OTf)_6$ in CD_3OD, [13.0 mM], 100 MHz.

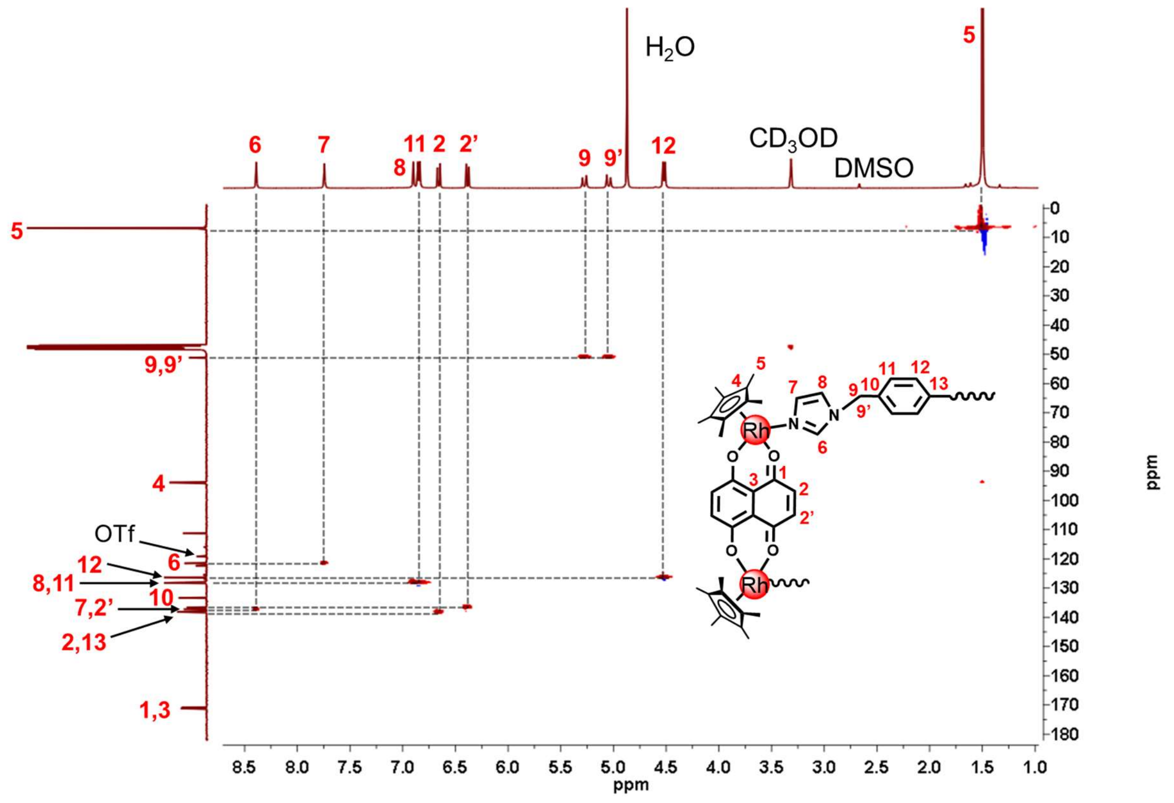

Figure S20. ^{1}H-^{13}C HSQC spectrum of [**3-TK**]$(OTf)_6$ in CD_3OD [13.0 mM].

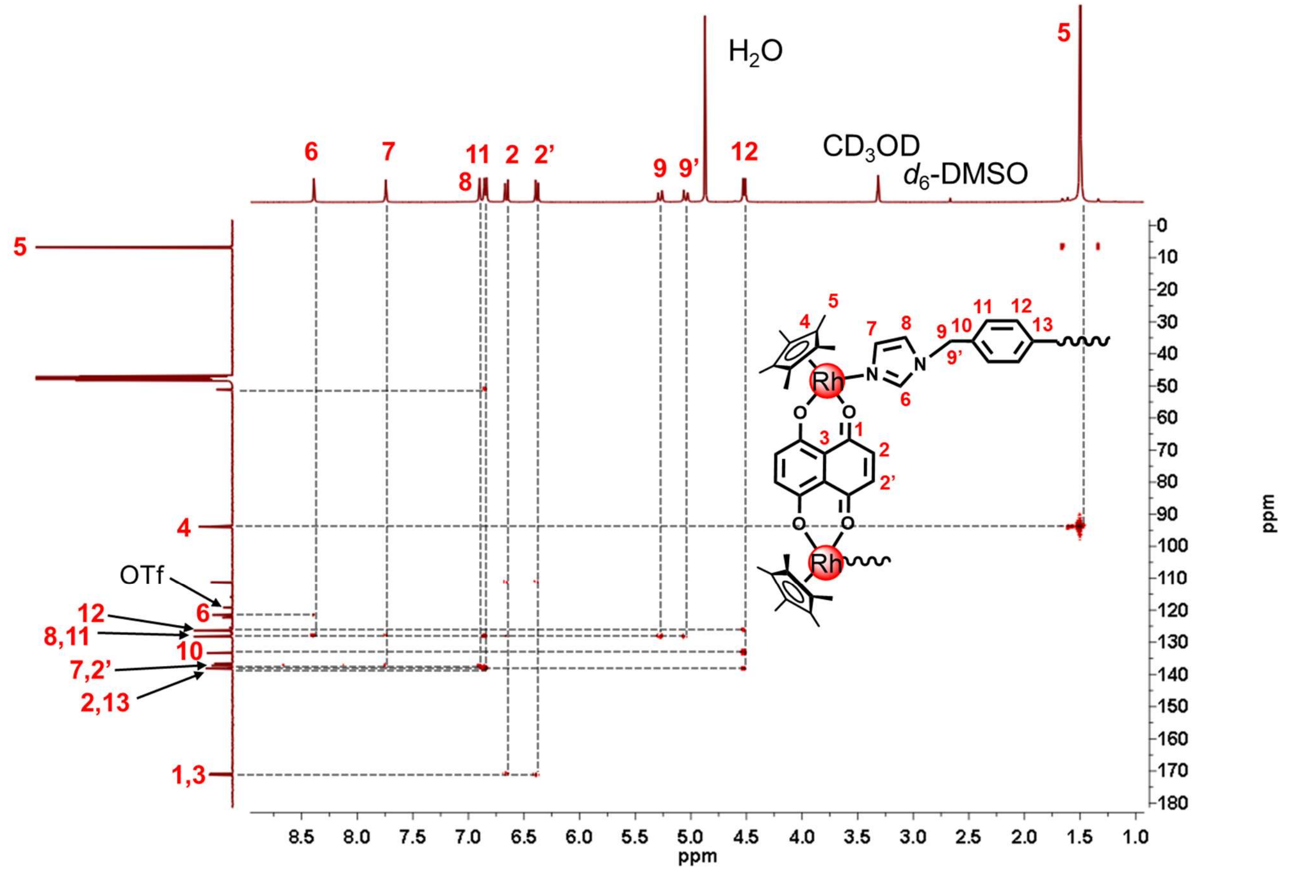

Figure S21. ^{1}H-^{13}C HMBC spectrum of [**3-TK**](OTf)$_6$ in CD$_3$OD, [13.0 mM].

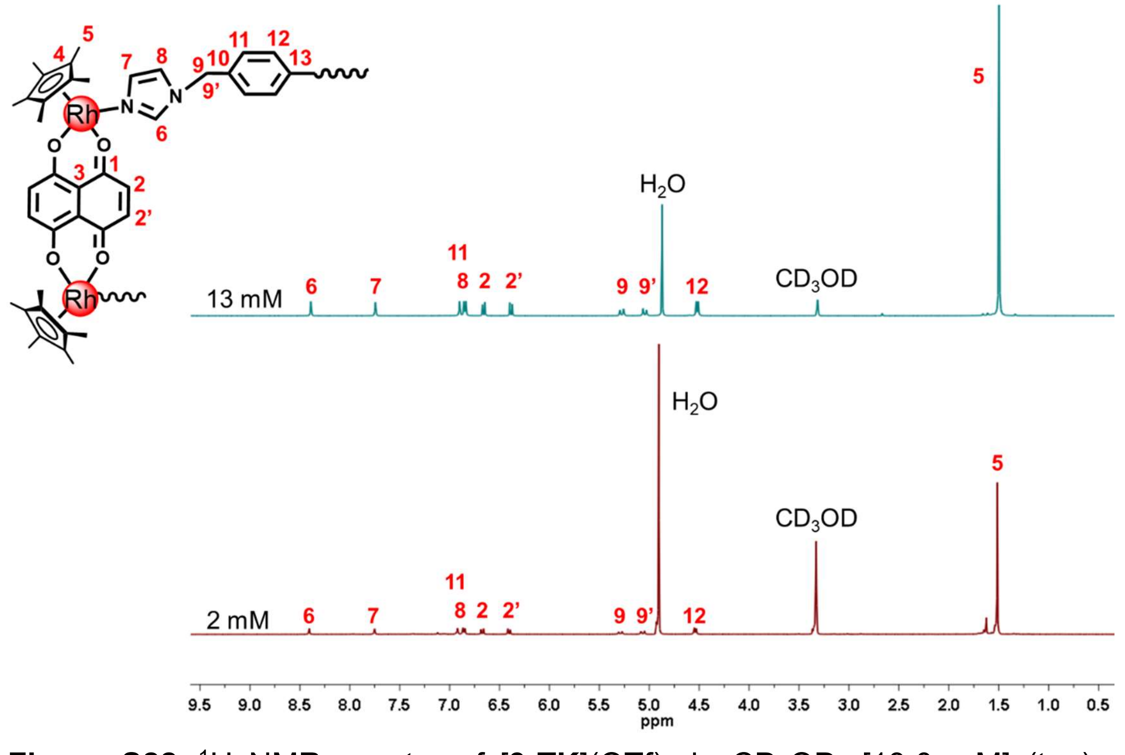

Figure S22. ^{1}H NMR spectra of [**3-TK**](OTf)$_6$ in CD$_3$OD, [13.0 mM] (top) and [2.0 mM] (bottom).

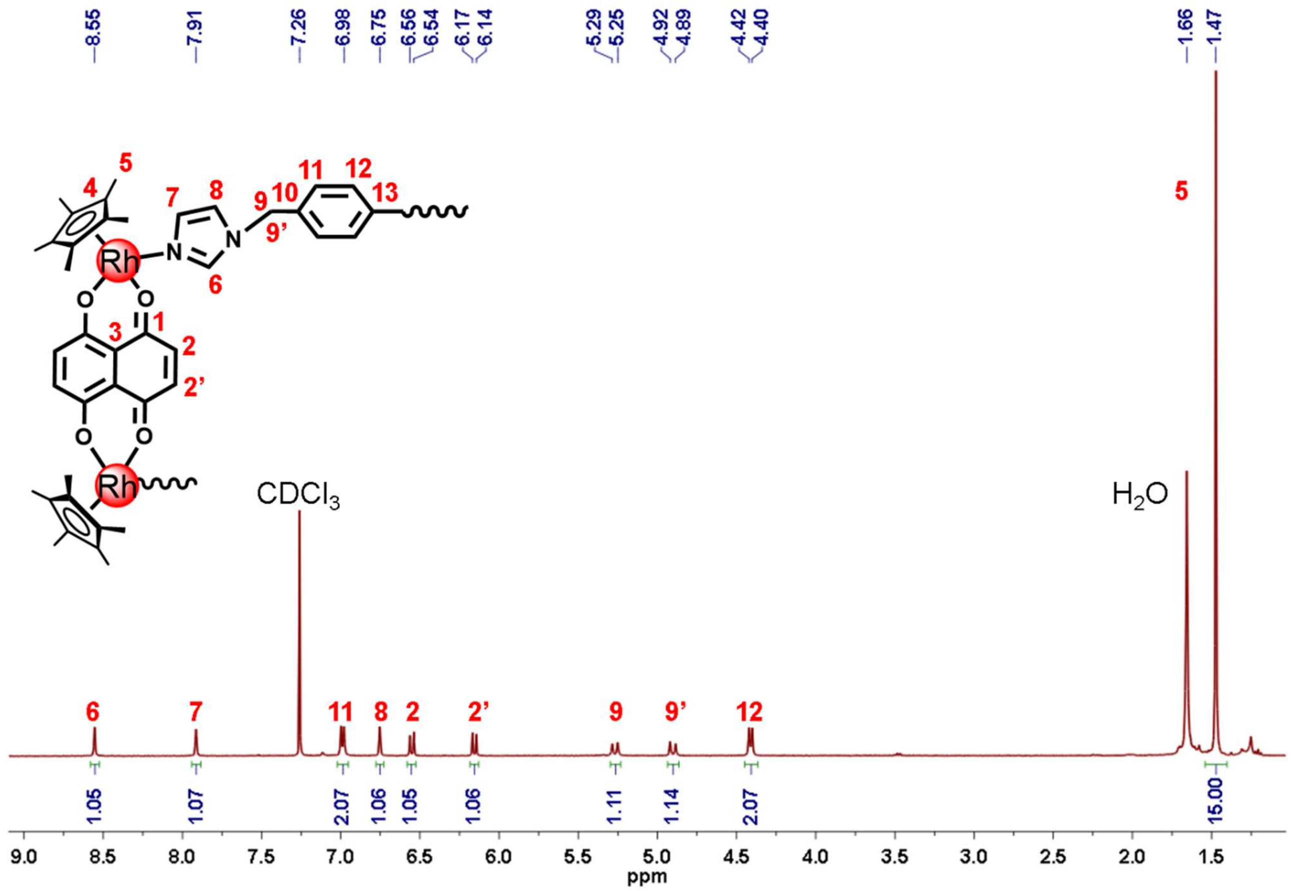

Figure S23. ^{1}H NMR spectrum of [**3-TK**](OTf)$_6$ in $CDCl_3$, [5.0 mM], 400 MHz.

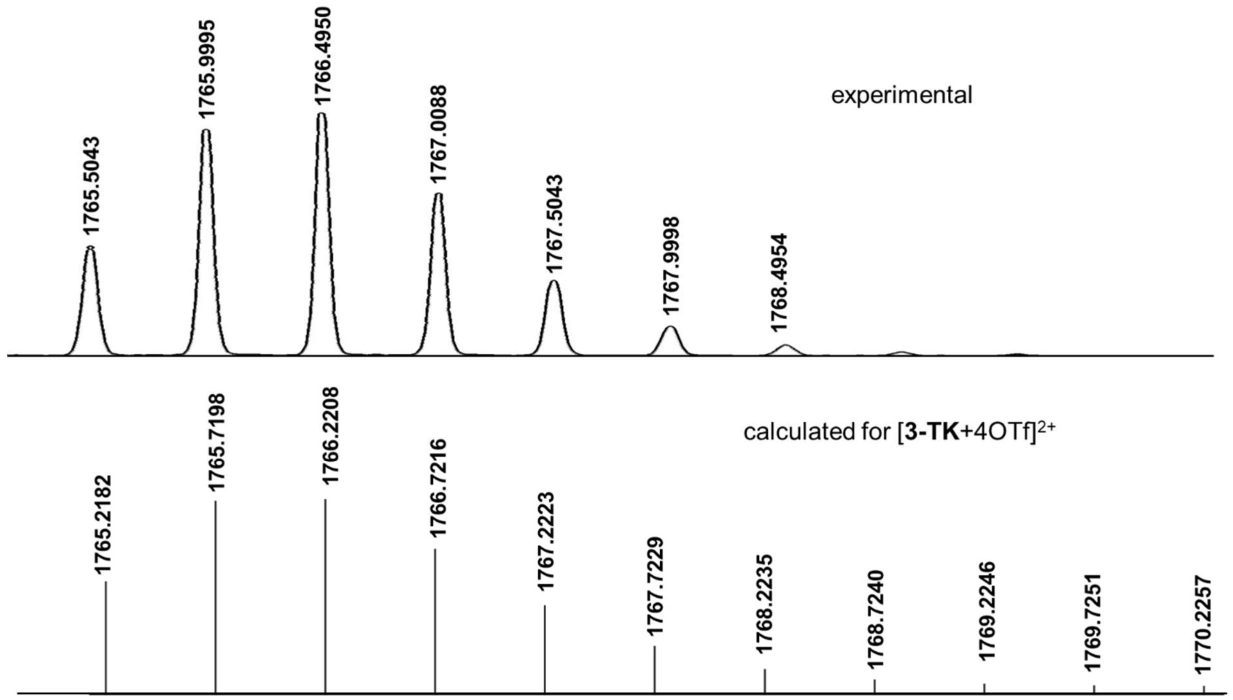

Figure S24. Section of the experimental (top) and calculated (bottom) ESI HRMS spectrum of [**3-TK**](OTf)$_6$.

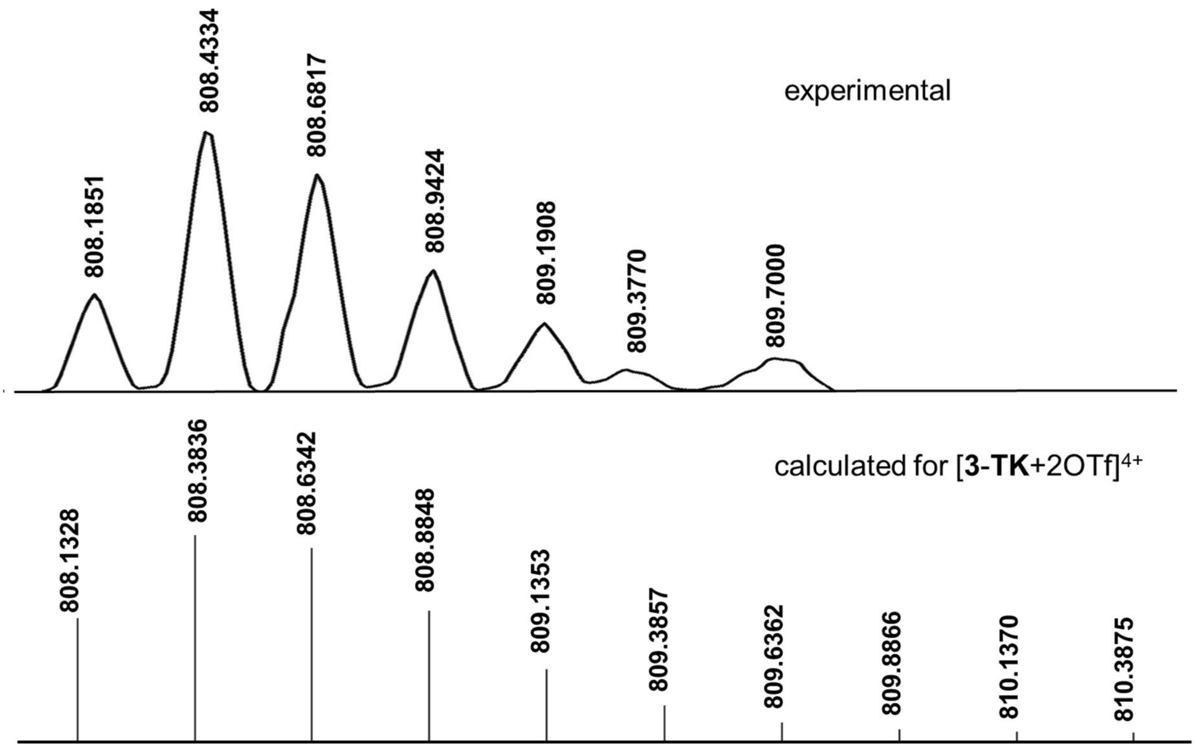

Figure S25. Section of the experimental (top) and calculated (bottom) ESI HRMS spectrum of [**3-TK**]$(OTf)_6$.

3. Ultraviolet-visible absorption spectra

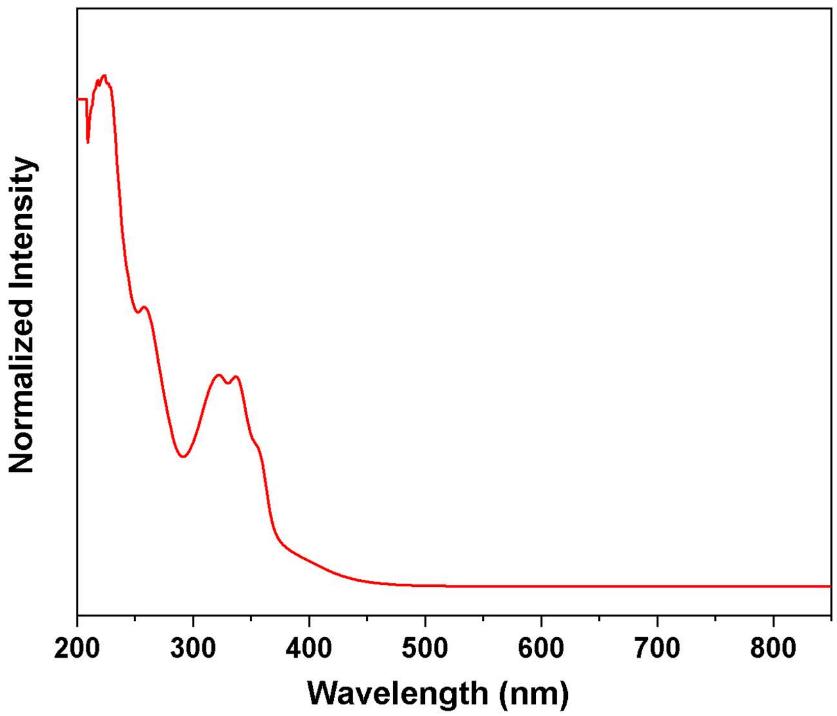

Figure S26. Ultraviolet-visible absorption spectrum of [**2**](OTf)$_2$ in methanol.

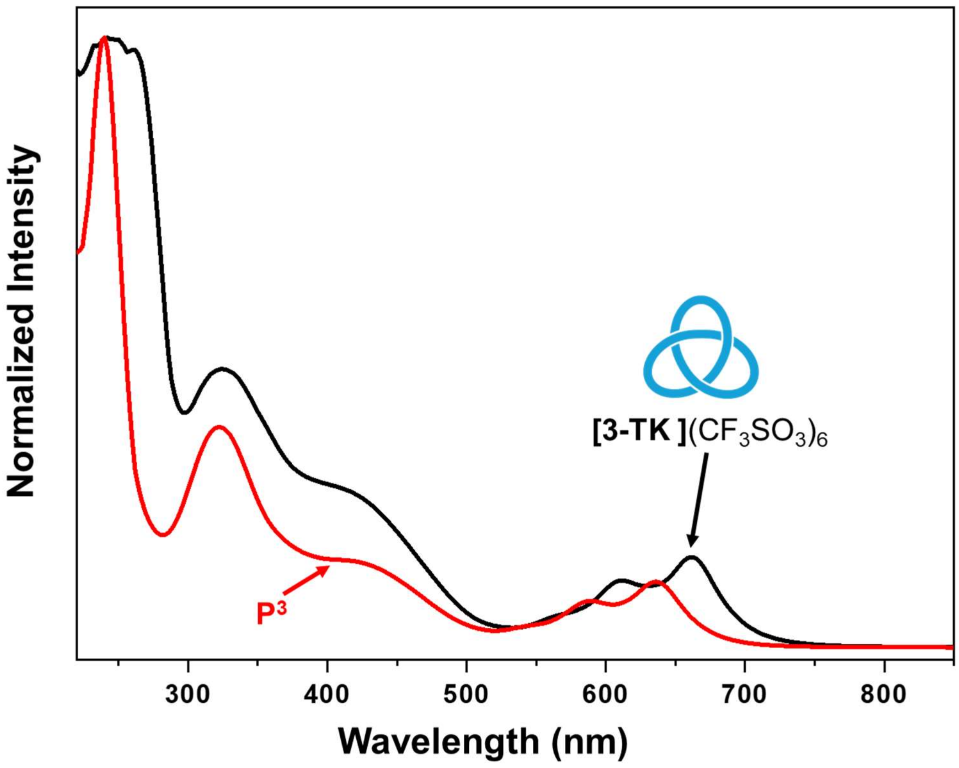

Figure S27. Ultraviolet-visible absorption spectra of [**3-TK**](CF_3SO_3)$_6$ and of building block **P^3** in methanol.

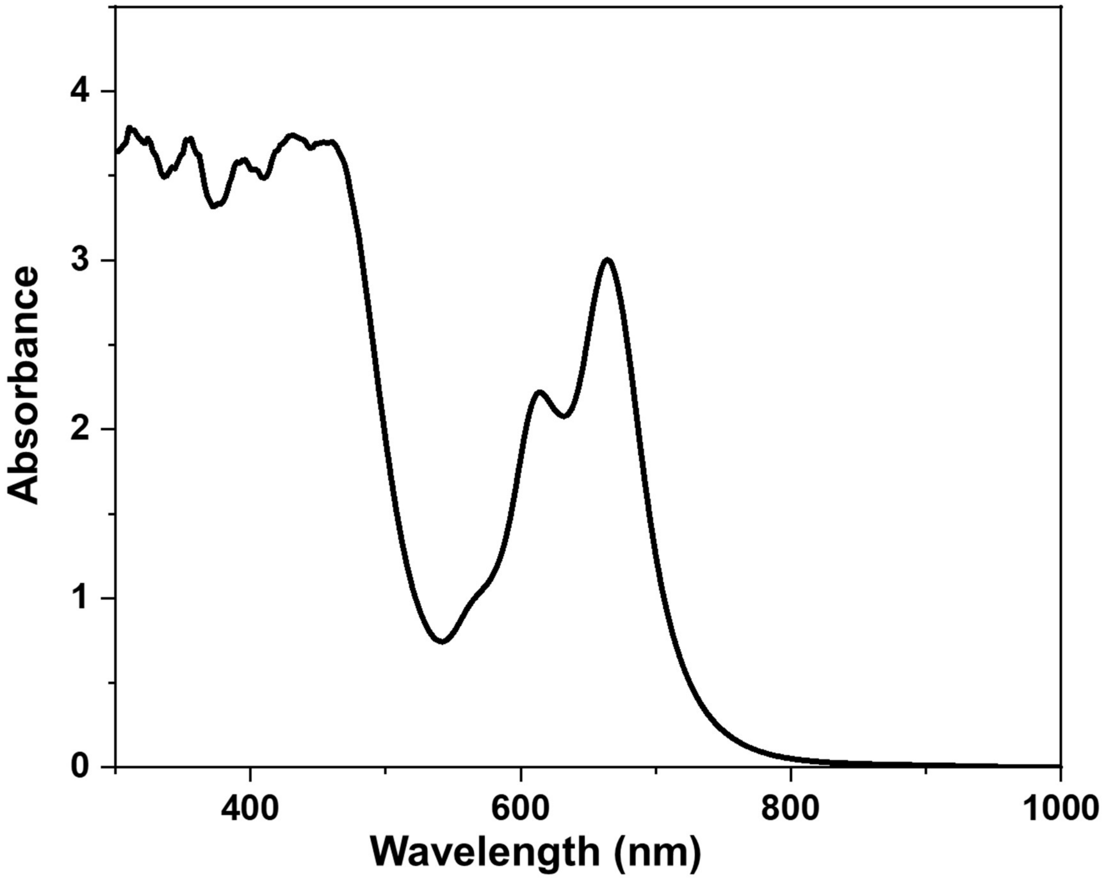

Figure S28. Ultraviolet-visible absorption spectrum of [**3-TK**]$(CF_3SO_3)_6$ (300 μM) in water (with 1% DMSO).

4. Photothermal performance

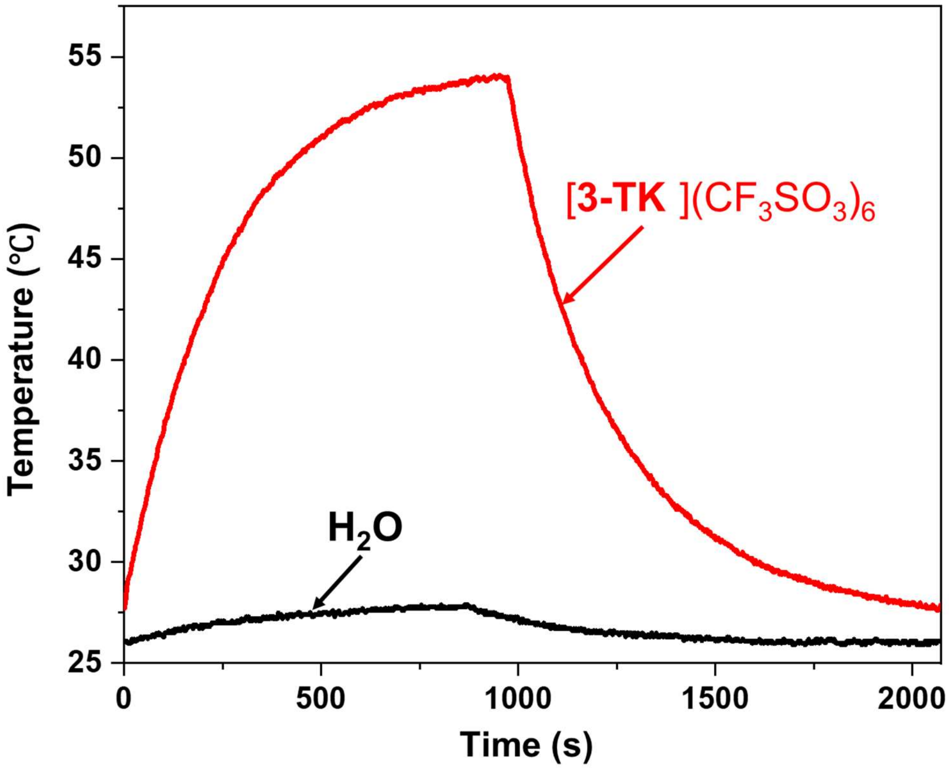

Figure S29. The heating and cooling rate curve of an aqueous solution of [**3-TK**](CF_3SO_3)$_6$ (300 μM, with 1% DMSO) and of water (with1% DMSO).

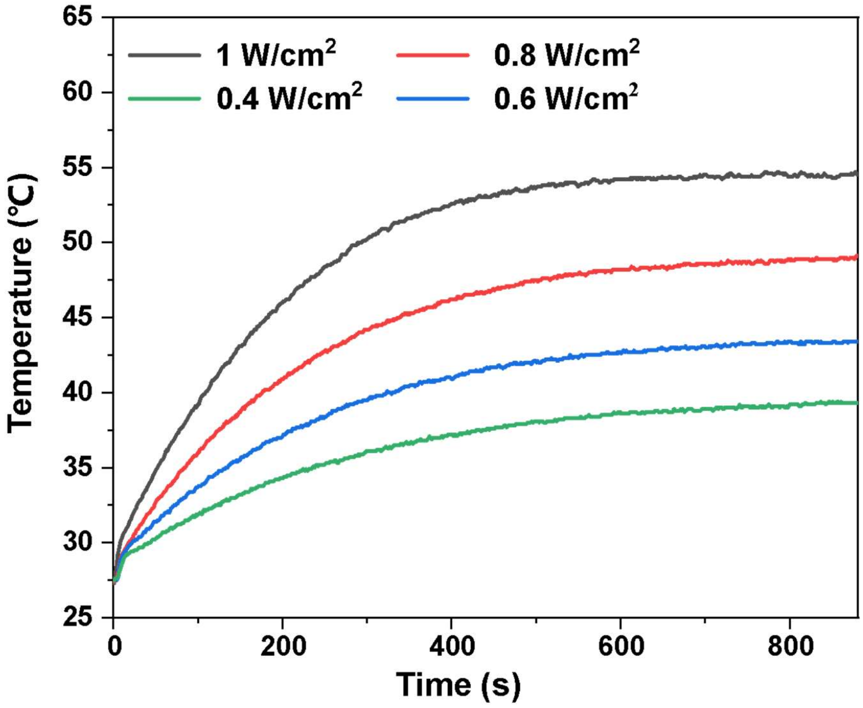

Figure S30. The heating curve of [**3-TK**](CF_3SO_3)$_6$ (300 μM) with different laser power densities under irradiation at 730 nm.

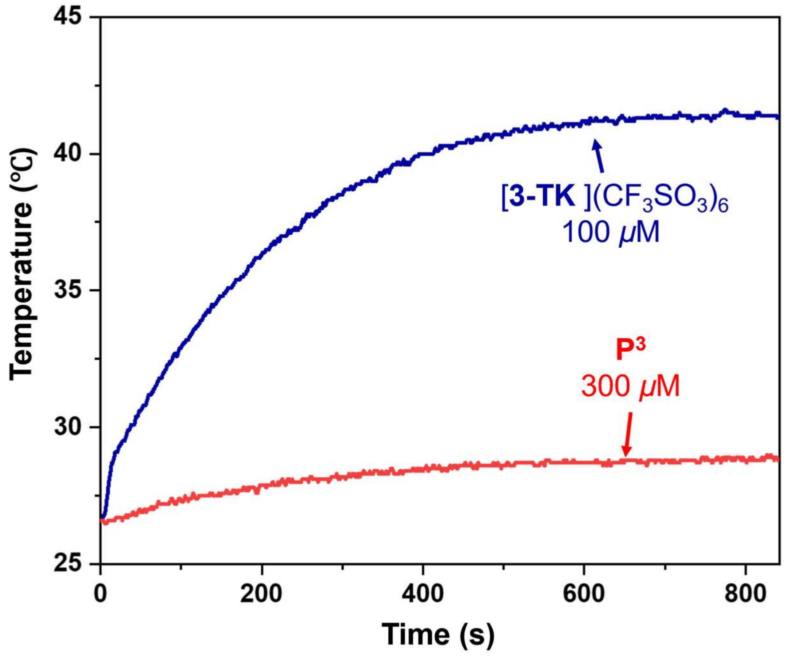

Figure S31. Comparison of the heating curve of aqueous solutions of [**3-TK**](CF_3SO_3)$_6$ (100 μM, with 1% DMSO) and building block **P**^{3} (300 μM, with 1% DMSO).

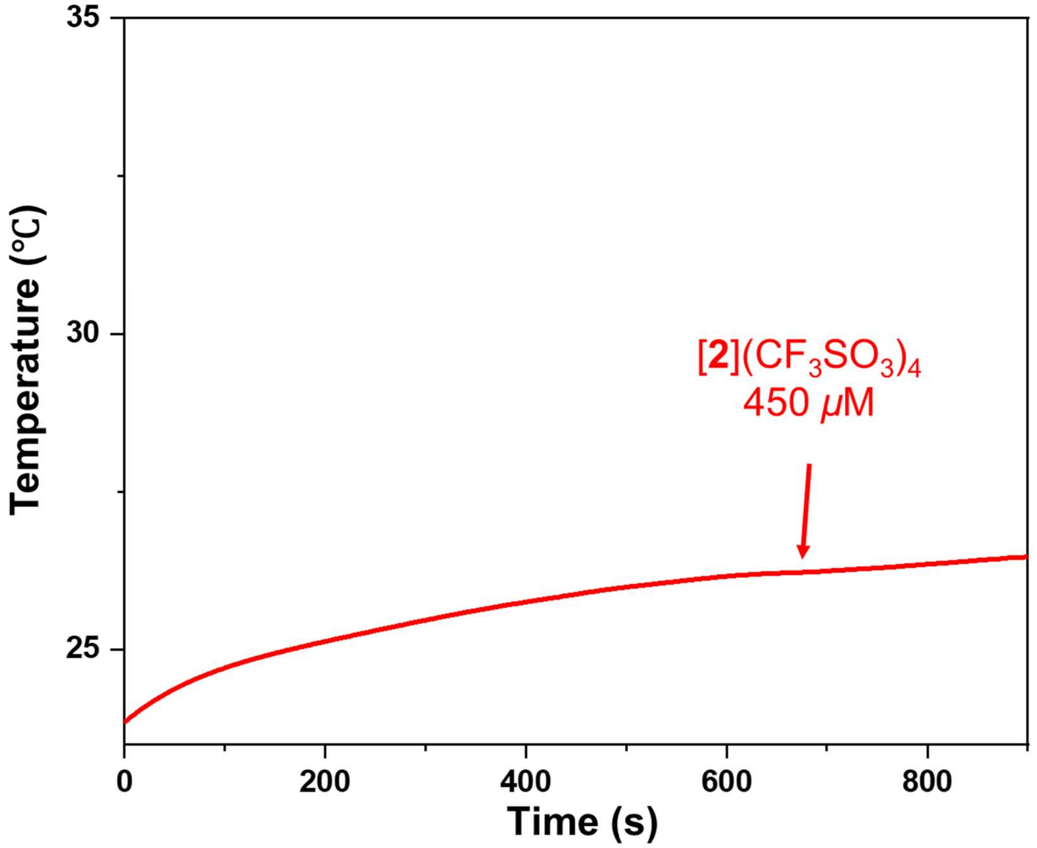

Figure S32. The heating curve of aqueous solutions of [**2**](CF_3SO_3)$_4$ (450 μM, with 1% DMSO) under irradiation at 730 nm.

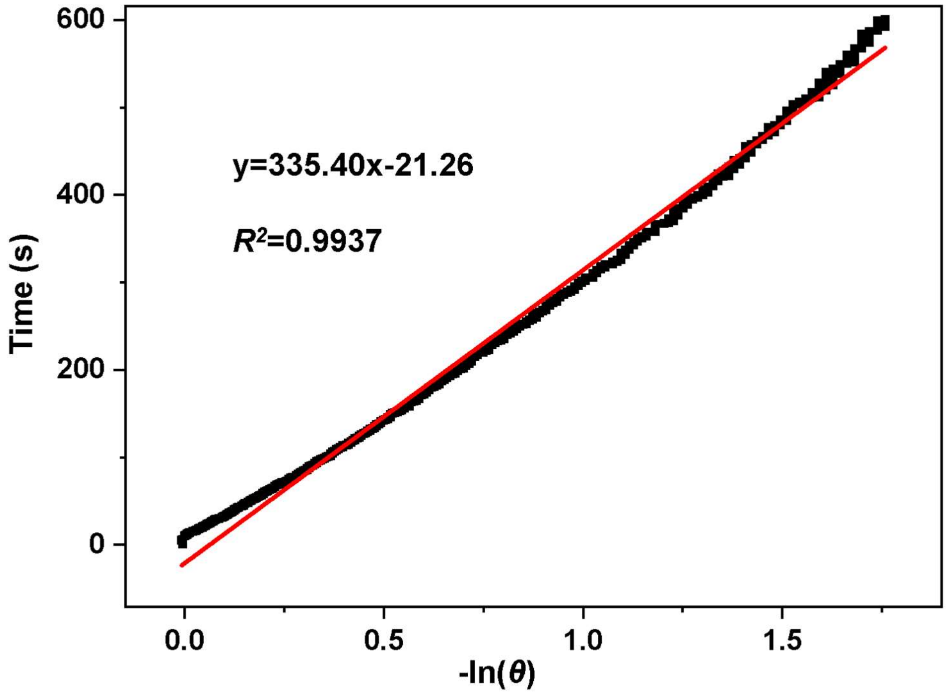

Figure S33. Fitting curve of time vs -ln(θ) of an aqueous solution of [**3-TK**]($CF_3SO_3)_6$ (300 μM, ln(θ) is the linear time data obtained from the cooling experiment in Figure S29).

5. Calculation of the photothermal conversion efficiency

The photothermal conversion efficiency (PCE) of [**3-TK**]$(CF_3SO_3)_6$ was calculated using the standard PCE calculation method:

$$\eta = \frac{h \cdot A\left[\left(T_{max(sample)} - T_{sur}\right) - T_{max(water)} - T_{sur}\right]}{I\left[1 - 10^{-A_{730}}\right]} \quad (1)$$

$$h \cdot A = \frac{m_{water} \cdot C_{water}}{\tau_s} \quad (2)$$

$$\tau_s = -\frac{t}{ln\theta} \quad (3)$$

$$\theta = \frac{T - T_{sur}}{T_{max} - T_{sur}} \quad (4)$$

Where *m* (g) is the mass of the aqueous solution, C_{water} is the specific heat capacity of water, T_{max} (°C) represents the maximum temperature of the solution, T_{sur} (°C) is the ambient temperature, *A* is the absorbance of the sample at 730 nm, T_s is the cooling time, and *T* (°C) is the temperature of the solution during cooling.

The calculation process is as follows:

$T_{max(sample)} = 54.3$ ℃ $\quad T_{max(water)} = 27.9$ ℃ $\quad T_{sur(sample)} = 26$ ℃

$A_{730} = 0.419 \quad C_{water} = 4.2 \times 10^3\ J \cdot Kg^{-1} \cdot ℃^{-1} \quad V_{water} = 0.5\ ml$

$I = 1\ W \cdot cm^2 \times \pi \times (0.34)^2 = 0.1156\,\pi\,W$

$$h \cdot A = \frac{m_{water} \cdot C_{water}}{\tau_s} = \frac{0.5 \times 10^{-3}\ Kg \cdot 4.2 \times 10^3\ J \cdot Kg^{-1} \cdot ℃^{-1}}{335.4\ s} = 6.26 \times 10^{-3}\ W \cdot ℃^{-1}$$

$$\eta = \frac{h \cdot A\left[\left(T_{max(sample)} - T_{sur}\right) - \left(T_{max(water)} - T_{sur}\right)\right]}{I\left[1 - 10^{-A_{730}}\right]}$$

$$= \frac{6.26 \times 10^{-3}\ W \cdot ℃^{-1} \times (54.3 - 27.9)℃}{0.1156\,\pi\,W \times (1 - 0.381)} = 73.5\%$$

6. Photoacoustic performance

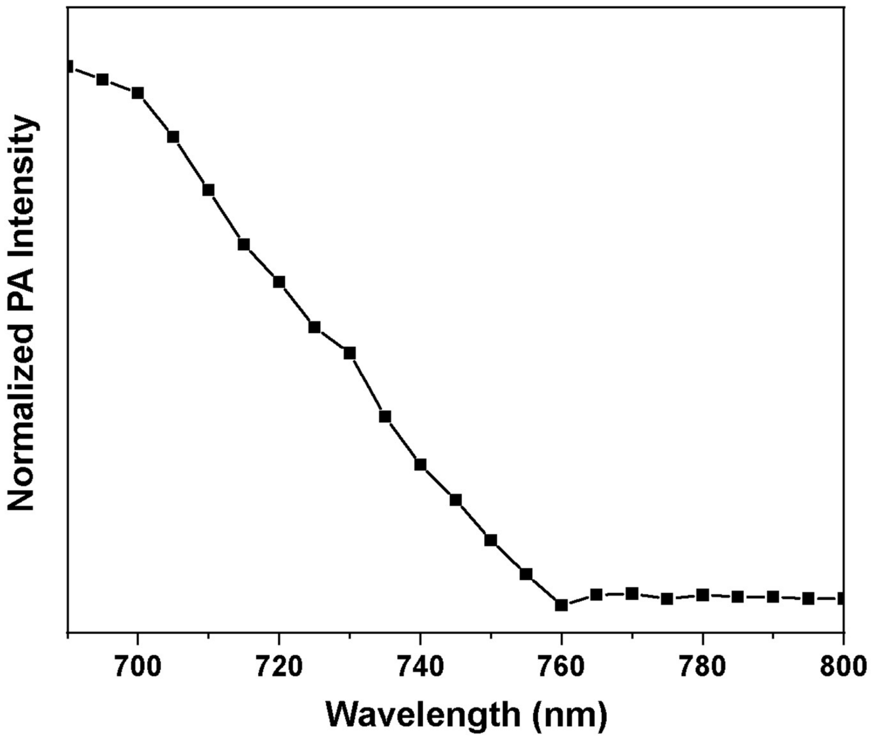

Figure S34. PA signal intensities of an aqueous solution of [**3-TK**](CF_3SO_3)$_6$ (300 μM, with 1% DMSO) at different wavelengths.

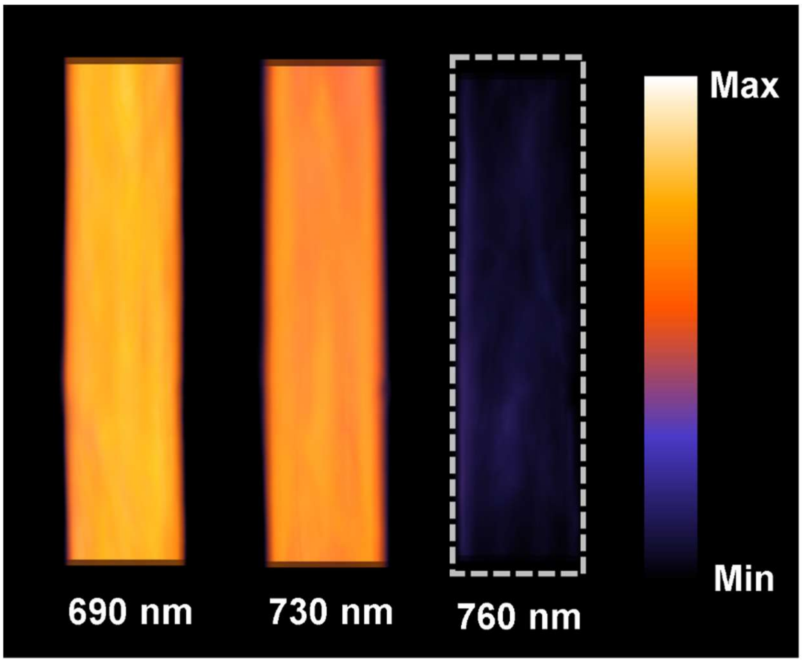

Figure S35. PA images of aqueous solutions of [**3-TK**](CF_3SO_3)$_6$ (300 μM, with 1% DMSO) at different wavelength.

7. X-ray diffraction studies

Yellow crystals of [**1**]$(CF_3SO_3)_2$·MeOH were obtained by slow diffusion of diethyl ether into a solution of the compound in methanol at ambient temperature. Orange crystals of [**2**]$CF_3SO_3)_4$·4MeOH·4H_2O were obtained by slow diffusion of diethyl ether into a concentrated methanol solution of the compound at ambient temperature. Green crystals of [**3-TK**]$(CF_3SO_3)_6$·DMSO·5MeOH·3H_2O were obtained by slow diffusion of diethyl ether into a concentrated methanol solution of the compound at ambient temperature. For the crystallization of both compounds, a small amount of DMSO or DMF (5%–10%) was added to improve crystallization and crystal quality.

Diffraction data for [**1**]$(CF_3SO_3)_2$·MeOH, [**2**]$(CF_3SO_3)_4$·4MeOH·4H_2O and [**3-TK**]$(CF_3SO_3)_6$·2DMSO·5MeOH·3H_2O were collected at 173(2) K with CCD-Bruker SMART APEX system (Ga-K_α, λ = 1.34138 Å). Indexing was performed using APEX 2 (difference vectors method). Data integration and reduction were performed using SaintPlus 6.01. Absorption correction was performed by the multiscan method implemented in SADABS. Structure solutions were found with SHELXT[S3] and were refined with SHELXL-97[S4] using first isotropic and later anisotropic thermal parameters for all non-hydrogen atoms.

The asymmetric unit of [**1**]$(CF_3SO_3)_2$·MeOH contains one formula unit. The asymmetric unit of [**2**]$(CF_3SO_3)_4$·4MeOH·4H_2O contains ½ formula unit related to the other half by a twofold axis. Severely disordered solvent molecules (one MeOH and two H_2O) were found in the asymmetric unit, which could not be restrained properly. Therefore, the SQUEEZE algorithm was used to omit them. One triflate anion, one bi-imidazole ligand and one Cp* fragment in the asymmetric unit were also disordered and they were divided into two parts (59:41 for the triflate anion, 56:44 for the biimidazole and 59:41 for the Cp*). 16 ISOR, 2 SIMU, 4 DANG and 22 DFIX instructions were used to restrain the anions, the ligand and the Cp* fragment leading to 404 restraints in the data. The disorder of the bisimidazole and the Cp* ligand did not significantly affect the overall geometry of the molecule. The OH hydrogen atom position of the located methanol molecule (at atom O7) was located in a Difference Fourier map and all other hydrogen atoms were placed on calculated positions.

The asymmetric unit of [**3-TK**]$(CF_3SO_3)_6$·2DMSO·5MeOH·3H_2O contains one formula unit.

Five of the six triflate anions, one DMSO molecule, the five methanol molecules and the three water molecules are strongly disordered and could not be restrained properly. The SQUEEZE algorithm was used to omit them. The trefoil knot was partly disordered and it was divided into two parts (59:41 for Rh5, Rh6, 66:34 for Rh2, Rh3 and 71:29 for the Cp* fragment). 191 ISOR, 1 FLAT, 7 SIMU, 8 DANG and 84 DFIX instructions were used to restrain anion, solvent molecule and the trefoil knot so that there are a total of 1468 restraints.

Crystallographic data for [**1**]$(CF_3SO_3)_2$·MeOH, [**2**]$(CF_3SO_3)_4$·4MeOH·$4H_2O$ and [**3-TK**]$(CF_3SO_3)_6$·2DMSO·5MeOH·$3H_2O$ have been deposited in the Cambridge Crystallographic Data Centre under accession numbers CCDC 2481824 for [**1**]$(CF_3SO_3)_2$·MeOH, CCDC 2481775 for [**2**]$(CF_3SO_3)_4$·4MeOH·$4H_2O$ and CCDC 2481793 for [**3-TK**]$(CF_3SO_3)_6$·2DMSO·5MeOH·$3H_2O$.

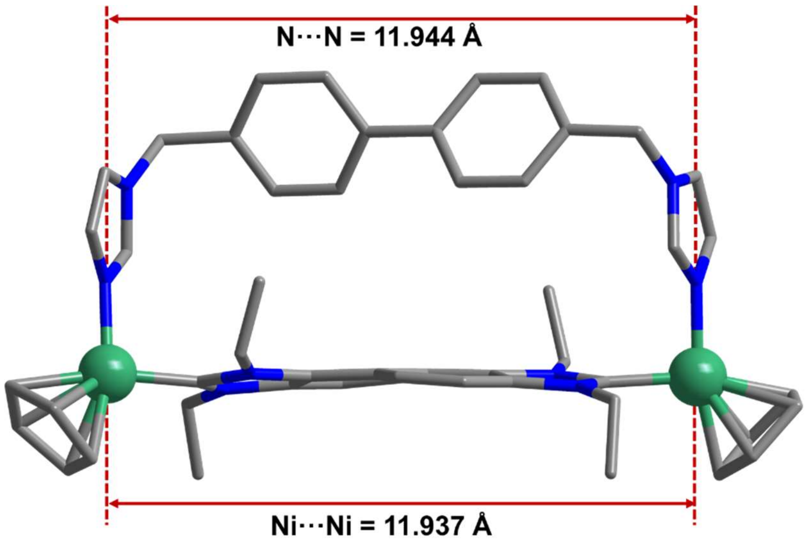

Figure S36. Molecular structure of complex cation $[\mathbf{1}]^{2+}$ in [**1**]$(CF_3SO_3)_2$·MeOH (color code: C, grey; N, blue; Ni, green; Rh, yellow; hydrogen atoms are omitted).

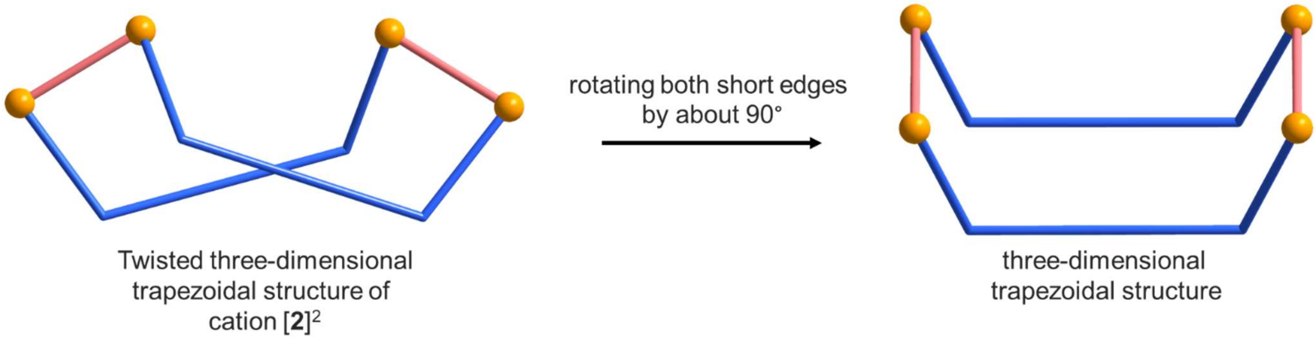

Figure S37. Structural representation of complex cation [**2**]$^{4+}$ in [**2**]$(CF_3SO_3)_4 \cdot 4MeOH \cdot 4H_2O$ (left) and trapezoidal structure which would result from rotating the short edges in parallel positions.

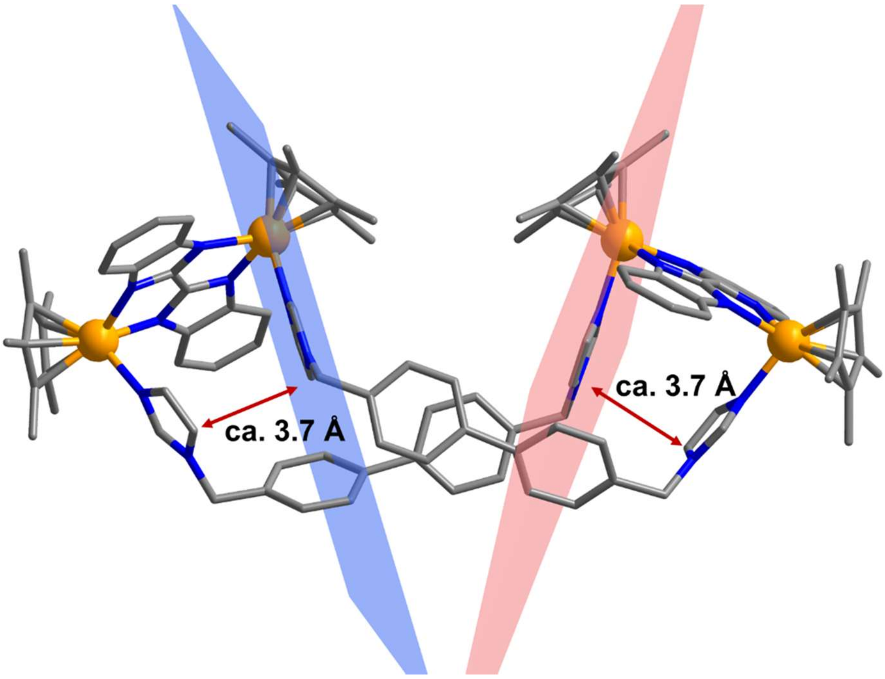

Figure S38. Molecular structure of cation [**2**]$^{4+}$ in [**2**]$(CF_3SO_3)_4 \cdot 4MeOH \cdot 4H_2O$ (color code: C, grey; N, blue; Rh, yellow; hydrogen atoms are omitted). The distance between two imidazole groups measures about 3.7 Å.

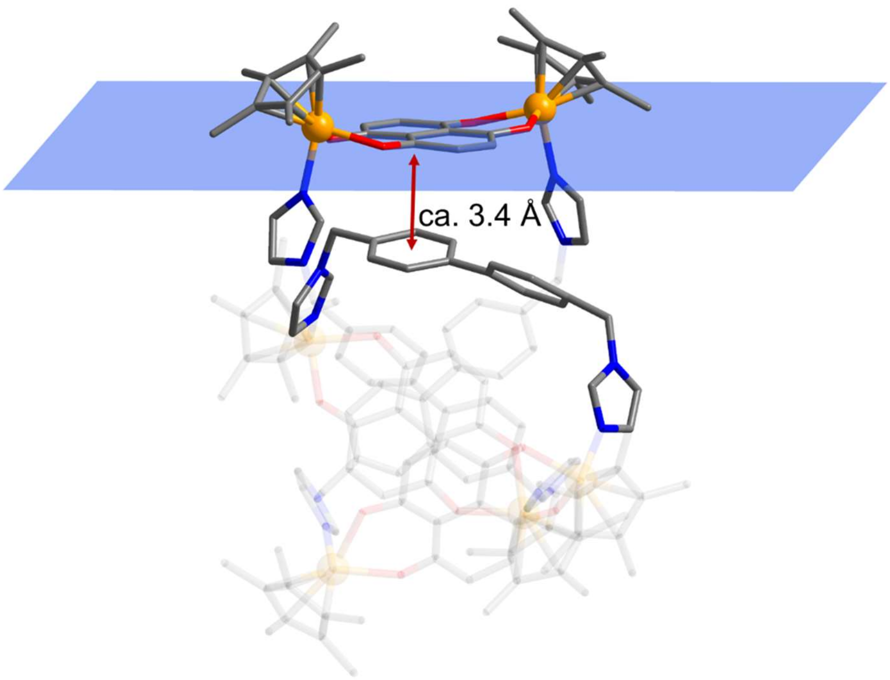

Figure S39. Molecular structure of cation [**3-TK**]$^{6+}$ in [**3-TK**]$(CF_3SO_3)_6$·2DMSO·5MeOH·$3H_2O$ (color code: C, grey; N, blue; Rh, yellow; hydrogen atoms are omitted). The distance between naphthalene planes of building block **P^3** and the biphenyl groups of ligand **L** measures about 3.4 Å.

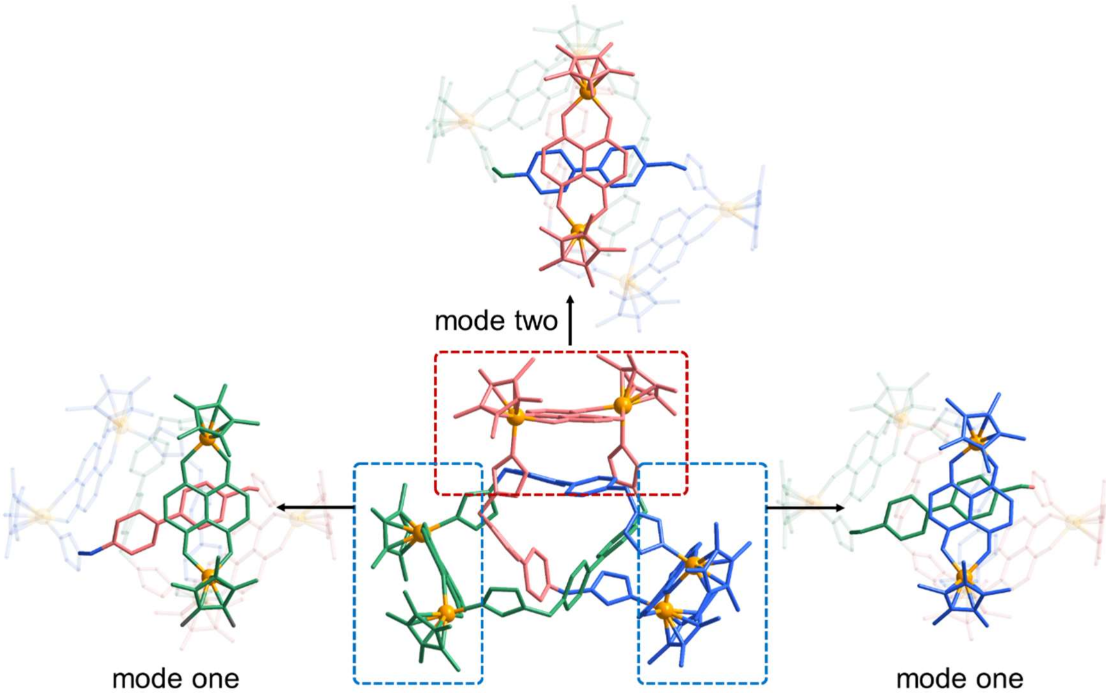

Figure S40. Molecular structure of trefoil knot [**3-TK**]$^{6+}$ with each leaf depicted in a different color (hydrogen atoms have been omitted). There are two distinctly different $\pi\cdots\pi$ stacking modes between the dinuclear complex **P^3** and the biphenyl units of ligand **L**. In mode one, the naphthalene group stacks over a single phenyl ring of the biphenyl unit (asymmetric), while in mode two, it stacks over the central biphenyl C_{ph}–C_{ph} bond (symmetric).

Table S1. Crystal data and structure refinement for [**1**](CF_3SO_3)$_2$·MeOH

Empirical formula	$C_{53}H_{56}N_8F_6Ni_2O_7S_2$
Formula weight	1212.59
Temperature/K	173(2)
Crystal system	triclinic
Space group	$P-1$
a/Å	13.4810(4)
b/Å	14.1801(4)
c/Å	15.8957(5)
α/°	109.2510(10)
β/°	97.2420(10)
γ/°	100.0030(10)
Volume/Å^3	2769.66(14)
Z	2
ρ_{calcd}/g·cm^{-3}	1.454
μ/mm^{-1}	4.612
F(000)	1256
Crystal size/mm^3	0.3 × 0.26 × 0.24
Radiation	GaKα (λ = 1.34139)
2Θ range for data collection/°	6.32 to 113.0
Index ranges	$-16 \le h \le 16$, $-17 \le k \le 17$, $-19 \le l \le 19$
Reflections collected	43462
Independent reflections	11078 [R_{int} = 0.0623]
Data/restraints/parameters	11078/72/793
Goodness-of-fit on F^2	1.127
Final R indexes [I>=2σ(I)]	R_1 = 0.0548, wR_2 = 0.1598
Final R indexes [all data]	R_1 = 0.0642, wR_2 = 0.1660
Largest diff. peak/hole/e·Å^{-3}	1.02/−0.73

$R_1 = \Sigma||F_0|-|F_c||$ (based on reflections with $F_0^2>2\sigma F^2$). $wR_2 = [\Sigma[w(F_0-F_c^2)^2]/\Sigma[w(F_0)^2]]^{1/2}$, $w=1/[\sigma^2(F_0^2)+(0.095P)^2]$, $P = [\max(F_0^2,0)+2F_c^2]/3$ (also with $F_0^2>2\sigma F^2$)

Table S2. Crystal data and structure refinement for [**2**]$(CF_3SO_3)_4 \cdot 4MeOH \cdot 4H_2O$

Empirical formula	$C_{116}H_{136}N_{16}F_{12}O_{20}Rh_4S_4$
Formula weight	2842.28
Temperature/K	173(2)
Crystal system	monoclinic
Space group	*C*2/*c*
a/Å	25.5980(7)
b/Å	15.3056(4)
c/Å	31.7533(9)
α/°	90
β/°	95.2970(10)
γ/°	90
Volume/Å^3	12387.6(6)
Z	4
ρ_{calc}/g·cm^{-3}	1.524
μ/mm^{-1}	3.782
F(000)	5824
Crystal size/mm^3	0.2 × 0.15 × 0.12
Radiation	GaKα (λ = 1.34139)
2Θ range for data collection/°	7.39 to 109.75
Index ranges	−31 ≤ h ≤ 31, −18 ≤ k ≤ 18, −38 ≤ l ≤ 38
Reflections collected	88979
Independent reflections	11727 [R_{int} = 0.0467]
Data/restraints/parameters	11727/404/998
Goodness-of-fit on F^2	1.044
Final *R* indexes [I>=2σ(I)]	R_1 = 0.0511, wR_2 = 0.1445
Final *R* indexes [all data]	R_1 = 0.0576, wR_2 = 0.1508
Largest diff. peak/hole/e·Å^{-3}	1.67/−0.91

$R_1 = \Sigma||F_0|-|F_c||$ (based on reflections with $F_0^2>2\sigma F^2$). $wR_2 = [\Sigma[w(F_0-F_c^2)^2]/\Sigma[w(F_0)^2]]^{1/2}$, $w=1/[\sigma^2(F_0^2)+(0.095P)^2]$, $P = [\max(F_0^2,0)+2F_c^2]/3$ (also with $F_0^2>2\sigma F^2$)

Table S3. Crystal data and structure refinement for [**3-TK**]$(CF_3SO_3)_6$·2DMSO·5MeOH·$3H_2O$

Empirical formula	$C_{165}H_{194}N_{12}F_{18}O_{40}Rh_6S_8$
Formula weight	4201.25
Temperature/K	173(2)
Crystal system	triclinic
Space group	*P*−1
a/Å	16.4343(12)
b/Å	18.6445(18)
c/Å	31.8791(19)
α/°	80.330(3)
β/°	83.069(2)
γ/°	74.834(3)
Volume/Å^3	9263.6(13)
Z	2
ρ_{calc}/g·cm^{-3}	1.506
μ/mm^{-1}	3.937
F(000)	4296.0
Crystal size/mm^3	0.15 × 0.13 × 0.1
Radiation	GaKα (λ = 1.34139)
2Θ range for data collection/°	6.06 to 105.94
Index ranges	-19 ≤ h ≤ 19, -22 ≤ k ≤ 22, -37 ≤ l ≤ 37
Reflections collected	146298
Independent reflections	32552 [R_{int} = 0.0579]
Data/restraints/parameters	32552/1468/2456
Goodness-of-fit on F^2	1.037
Final *R* indexes [I>=2$\sigma(I)$]	R_1 = 0.0952, wR_2 = 0.2710
Final *R* indexes [all data]	R_1 = 0.1112, wR_2 = 0.2857
Largest diff. peak/hole / e Å^{-3}	1.67/−1.18

$R_1 = \Sigma||F_0|-|F_c||$ (based on reflections with $F_0^2>2\sigma F^2$). $wR_2 = [\Sigma[w(F_0-F_c^2)^2]/\Sigma[w(F_0)^2]]^{1/2}$, $w=1/[\sigma^2(F_0^2)+(0.095P)^2]$, $P = [\max(F_0^2,0)+2F_c^2]/3$ (also with $F_0^2>2\sigma F^2$)

8. References

S1. Y. W. Zhang, Y. Lu, L. Y. Sun, P. D. Dutschke, M. M. Gan, L. Zhang, A. Hepp, Y. F. Han, F. E. Hahn, *Angew. Chem. Int. Ed.* **2023**, *62*, e202312323; *Angew. Chem*. **2023**, *135*, e202312323.

S2. J. Chen, J. J. Xie, X. Y. Chen, R. Dong, X. H. Ge, T. Qiu *Green Chem.* **2024**, *26*, 10500–10511.

S3. G. M. Sheldrick, *Acta. Cryst*. **2015**, *A71*, 3–8.

S4. G. M. Sheldrick, *Acta. Cryst.* **2015**, *C71*, 3–8.
